# The Tongue Squamous Carcinoma Cell Line Cal27 Primarily Employs Integrin α6β4-Containing Type II Hemidesmosomes for Adhesion Which Contribute to Anticancer Drug Sensitivity

**DOI:** 10.3389/fcell.2021.786758

**Published:** 2021-12-16

**Authors:** Ana Tadijan, Jonathan D. Humphries, Ivana Samaržija, Nikolina Stojanović, Junzhe Zha, Kristina Čuljak, Marija Tomić, Mladen Paradžik, Davor Nestić, Heemin Kang, Martin J. Humphries, Andreja Ambriović-Ristov

**Affiliations:** ^1^ Laboratory for Cell Biology and Signalling, Division of Molecular Biology, Ruđer Bošković Institute, Zagreb, Croatia; ^2^ Laboratory for Protein Dynamics, Division of Molecular Medicine, Ruđer Bošković Institute, Zagreb, Croatia; ^3^ Wellcome Centre for Cell-Matrix Research, Faculty of Biology, Medicine and Health, Manchester Academic Health Science Centre, University of Manchester, Manchester, United Kingdom; ^4^ Department of Life Sciences, Manchester Metropolitan University, Manchester, United Kingdom; ^5^ Laboratory for Epigenomics, Division of Molecular Medicine, Ruđer Bošković Institute, Zagreb, Croatia; ^6^ Department of Materials Science and Engineering, Korea University, Seoul, South Korea

**Keywords:** adhesome, hemidesmosome, integrin alpha 6 beta 4, laminin-332, integrin alpha v beta 3, integrin crosstalk, anticancer drug sensitivity, keratins 5/14

## Abstract

Integrins are heterodimeric cell surface glycoproteins used by cells to bind to the extracellular matrix (ECM) and regulate tumor cell proliferation, migration and survival. A causative relationship between integrin expression and resistance to anticancer drugs has been demonstrated in different tumors, including head and neck squamous cell carcinoma. Using a Cal27 tongue squamous cell carcinoma model, we have previously demonstrated that *de novo* expression of integrin αVβ3 confers resistance to several anticancer drugs (cisplatin, mitomycin C and doxorubicin) through a mechanism involving downregulation of active Src, increased cell migration and invasion. In the integrin αVβ3 expressing Cal27-derived cell clone 2B1, αVβ5 expression was also increased, but unrelated to drug resistance. To identify the integrin adhesion complex (IAC) components that contribute to the changes in Cal27 and 2B1 cell adhesion and anticancer drug resistance, we isolated IACs from both cell lines. Mass spectrometry (MS)-based proteomics analysis indicated that both cell lines preferentially, but not exclusively, use integrin α6β4, which is classically found in hemidesmosomes. The anticancer drug resistant cell clone 2B1 demonstrated an increased level of α6β4 accompanied with increased deposition of a laminin-332-containing ECM. Immunofluorescence and electron microscopy demonstrated the formation of type II hemidesmosomes by both cell types. Furthermore, suppression of α6β4 expression in both lines conferred resistance to anticancer drugs through a mechanism independent of αVβ3, which implies that the cell clone 2B1 would have been even more resistant had the upregulation of α6β4 not occurred. Taken together, our results identify a key role for α6β4-containing type II hemidesmosomes in regulating anticancer drug sensitivity.

## Introduction

Integrins are transmembrane cell adhesion receptors consisting of α and β subunits. Eighteen α and eight β subunits are present in mammals, which can assemble to form 24 different dimers (Hynes, 2002). During tumorigenesis, the integrin repertoire is often switched to enable proliferation, survival, migration and invasion (Desgrosellier and Cheresh, 2010; Cooper and Giancotti, 2019; Samaržija et al., 2020). Cell adhesion is also associated with resistance to anticancer drugs (Damiano et al., 1999; Desgrosellier and Cheresh, 2010; Seguin et al., 2015; Dickreuter and Cordes, 2017). In head and neck squamous cell carcinoma (HNSCC), expression of several integrins, including αVβ3 and/or αVβ5, is increased compared to normal epithelium (Ahmedah et al., 2017). Changes in integrin expression have been observed in clones of human laryngeal carcinoma HEp2 cells produced by acute (Ambriović-Ristov et al., 2004) or chronic (Majhen et al., 2014) exposure to cisplatin (cDDP), respectively. A causative relationship between *de novo* expression of integrin αVβ3 and cDDP, mitomycin C (MMC) or doxorubicin (DOX) resistance through increased total amount of glutathione, which conferred survival due to better elimination of reactive oxygen species induced by anticancer drugs, has been demonstrated in the same model of HEp2 cells (Brozović et al., 2008). Similar effects have been reported in the HNSCC model Cal27 (tongue squamous carcinoma cells), although the mechanism of cDDP, MMC and DOX resistance was not the same, i.e., *de novo* expression of integrin αVβ3 conferred anticancer drug resistance through deactivation of Src (Stojanović et al., 2016). De novo expression of integrin αVβ3 in both cell models resulted in increased migration and invasion (Brozović et al., 2008; Stojanović et al., 2016).

Integrins bind to ECM proteins and, upon clustering, trigger signaling pathways through recruitment of multimolecular integrin adhesion complexes (IACs) to their cytoplasmic tails and through association with the cell cytoskeleton (Winograd-Katz et al., 2014). Integrins, together with associated IAC components, have been termed the adhesome (Zaidel-Bar et al., 2007; Kuo et al., 2011; Schiller et al., 2011; Byron et al., 2015; Jones et al., 2015; Chastney et al., 2020). Functional and morphological analyses have defined several major forms of IACs, including focal complexes, focal adhesions (FAs), fibrillar adhesions, hemidesmosomes (HDs) and reticular adhesions, also called clathrin lattices or plaques (Zuidema et al., 2020). FAs are strongly associated with actin filaments that link neighboring structures (Albiges-Rizo et al., 2009). Fibrillar adhesions are long, stable structures that run parallel to bundles of fibronectin (FN) *in vivo* and are highly enriched in tensin and α5β1 integrin (Zamir et al., 1999), while reticular adhesions are formed by integrin αVβ5 in the absence of both talin and F-actin (Lock et al., 2018, 2019). HDs facilitate stable adhesion of basal epithelial cells to the underlying basement membrane (BM) via integrin *α*6*β*4 and are associated with keratin intermediate filaments. The classical type I HDs consist of the integrin *α*6*β*4, plectin (PLEC; isoform 1a), tetraspanin CD151, BP230 (known as dystonin or BPAG1-e) and BP180 (known as BPAG2 or collagen XVII) (Walko et al., 2015), while type II HDs consist of only *α*6*β*4 and plectin (Uematsu et al., 1994; Fontao et al., 1999).

The ventral IACs from 2D cell cultures have been isolated using several protocols (Humphries et al., 2009; Kuo et al., 2011; Schiller et al., 2011; Jones et al., 2015) and analyzed by MS. However, most IAC preparations have been isolated from cells seeded on FN, which led to the definition of an FN-induced meta adhesome composed of over 2,400 proteins. Subsequent reduction defined 60 core proteins, termed the consensus adhesome (Winograd-Katz et al., 2014; Horton et al., 2015). There is less information on the adhesome of cells cultured on dishes without prior coating with ECM proteins. Lock et al. (2018) analyzed IACs of osteosarcoma U2OS cells cultured for 72 h and demonstrated that the most abundant integrin subunits were αV and β5, with much lower levels of β1, β3, β8, α5 and α3. Using a similar approach, we have recently analyzed the adhesome of the melanoma cell line MDA-MB-435S and found that these cells preferentially use integrin αVβ5 for adhesion during *in vitro* cultivation, forming either FAs or RAs (Paradžik et al., 2020). MS-based IAC analysis has an advantage over other adhesome analysis methods because it enables the simultaneous identification of ECM proteins secreted by cells and may therefore enable the identification of integrin-ECM interactions.

We have previously demonstrated that *de novo* expression of integrin αVβ3 in Cal27 cells confers resistance to cDDP, MMC and DOX through Src deactivation and contributes to increased migration and invasion. De novo expression of integrin αVβ3 caused an integrin crosstalk event, i.e., upregulation of integrin αVβ5 expression through increased production of integrin β5 mRNA. Integrin αVβ5 was not involved in the mechanism of anticancer drug resistance (Stojanović et al., 2016). To extend our understanding of the adhesion components and complexes whose differential expression contributes to the observed changes in adhesion and anticancer drug resistance, we isolated IACs from Cal27 cells and the Cal27-derived cell clone 2B1, with *de novo* expression of integrin αVβ3 and increased levels of αVβ5. MS analysis of isolated IACs showed that both cell lines preferentially, but not exclusively, use integrin α6β4. We showed that integrin α6β4 in Cal27 and 2B1 cells form type II HDs consisting of integrin α6β4 and PLEC. Moreover, in 2B1 cells, MS analysis revealed increased abundance of integrin α6β4 and PLEC, but decreased abundance of keratins KRT-5 and KRT-14 suggesting that these type II HDs have reduced anchorage to keratins. In addition, 2B1 cells demonstrated increased expression of BM components, i.e., integrin α6β4 receptor laminin-332 and collagen VII (COL7A1) which supports our conclusion of increased anchorage of cells via type II HDs. Finally, we showed that integrin β4 knockdown in Cal27 and 2B1 cells decreased expression of the integrin α6β4 heterodimer on the cell surface and conferred resistance to cDDP, MMC and DOX, thus showing an independent resistance mechanism than the one triggered by *de novo* expression of integrin αVβ3. This work contributes to the understanding of both the close connection between FAs and HDs in HNSCC cells and to the diversity of HD composition able to regulate sensitivity to anticancer drugs.

## Materials and Methods

### Cells

The human tongue squamous cell carcinoma cell line Cal27 was obtained from the American Type Culture Collection (ATCC, United States). Integrin αVβ3-expressing cell clone 2B1 was established from Cal27 cells by stable transfection with the pcDNAβ3 containing integrin subunit β3 cDNA (kindly provided by E. H. Danen, Leiden, Netherlands) as described (Stojanović et al., 2016). Cells were grown in Dulbecco’s modified Eagle’s medium (DMEM; Invitrogen, United States) supplemented with 10% (v/v) fetal bovine serum (FBS; Invitrogen, United States) at 37°C with 5% CO_2_ (v/v) in a humidified atmosphere.

### Survival Analysis

The anticancer drugs cDDP, MMC and DOX (Sigma-Aldrich, Germany) were dissolved in water and stored at −20°C. MTT (3-(4,5-dimethylthiazol-2-yl)-2,5-diphenyltetrazolium bromide, Millipore, United States) assay was used to determine the sensitivity of cells to anticancer drugs. Briefly, cells were treated 24 h after seeding in 96-well tissue culture plates (5 × 10^3^ cells/well). Cells previously transfected with siRNA were seeded 24 h upon transfection. Seventy-two hours upon anticancer drug exposure, the absorbance of MTT-formazan product dissolved in DMSO, which is proportional to the number of viable cells, was measured with a microplate reader (Awareness Technology, Inc., United States) at 600 nm.

### siRNA Transfection

For transient siRNA transfection experiments, cells were transfected with 25 nM siRNA specific for integrin subunit β4 (target sequence: GCG​ACT​ACA​CTA​TTG​GAT​T, Sigma, Germany) by Lipofectamine RNAiMax (Thermo Fisher Scientific, United States). Transfection efficacy was validated by sodium dodecyl sulphate-polyacrylamide-gel electrophoresis (SDS-PAGE) and western blot (WB) using integrin subunit β4-specific antibody and matching, labelled secondary antibodies (listed in Supplementary Table S1).

### Immunofluorescence and Confocal Microscopy

Cal27 and 2B1 cells were plated on coverslips at density of 3.5 × 10^4^ cells per well in a 24-well plate. After 48 h, cells were fixed with 4% (w/v) paraformaldehyde, permeabilized with 0.1% (v/v) Triton X-100, and the immunostaining was performed with the appropriate antibodies for 1 h, followed by incubation with conjugated secondary antibodies for 1 h. All antibodies are listed in Supplementary Table S1. Cells were mounted with DAPI Fluoromount-G (SouthernBiotech, United States). Fluorescence and respective IRM z-stack images (starting from the cell ventral surface) were acquired using an inverted confocal microscope (Leica TCS SP8 X, Leica Microsystems, Germany) with the HC PL APOCS2 63 ×/1.40 oil-immersion objective, zoom set at ×2.15. LAS X 3.1.1 (Leica Microsystems, Germany) software was used to analyze the images.

### Isolation of IACs, Sample Preparation for MS and Data Analysis

Integrin adhesion complexes were isolated as previously described (Jones et al., 2015; Paradžik et al., 2020). For each cell line, five biological replicates were analyzed. In short, cells (1 × 10^6^ for Cal27 and 9,4 × 10^5^ for 2B1) were plated on 10 cm diameter cell culture dishes (at least six dishes per cell line) and after 72 h washed with DMEM-HEPES and incubated with Wang and Richard’s reagent for 10 min (6 mM DTBP, Thermo Fisher Scientific, United States). DTBP was quenched by 0.03 M Tris-HCl (pH 8) and cells were lysed using modified RIPA buffer (50 mM Tris-HCl, pH 7.6; 150 mM NaCl; 5 mM disodium EDTA, pH 8; 1% (w/v) Triton X-100, 2.5% (w/v) SDS, 1% (w/v) sodium deoxycholate). Cell bodies were removed by high-pressure washing and remaining adhesion complex components were collected by scraping into the adhesion recovery solution (125 mM Tris-HCl, pH 6.8; 1% (w/v) SDS; 150 mM dithiothreitol). Isolated IACs were acetone precipitated and processed for either MS or WB analysis. For MS analysis, samples were prepared using in-gel trypsin digestion (Jones et al., 2015; Paradžik et al., 2020), and analyzed using a modified version of the LC-MS/MS method, as previously described (Horton et al., 2015). Briefly, an UltiMateR 3000 Rapid Separation LC (RSLC, United States) coupled to an Orbitrap Elite mass spectrometer (Thermo Fisher Scientific, United States) with electrospray ionization was used. Peptide mixtures were eluted for 44 min using a gradient containing 92% of solution A (0.1% formic acid in water) and 8% up to 33% of solution B (0.1% formic acid in acetonitrile). Solvent flow was set to 300 nl per minute. To identify proteins, data were searched against the human Uniprot database (version 2018_01) using Mascot (Matrix science, version 2.5.1). Fragment ion tolerance was set to 0.50 Da and parent ion tolerance was 5 PPM. Protein identifications were further refined using Scaffold (Proteome software). Protein (99%) and peptide (90%) probabilities were assigned using the Protein Prophet algorithm (Nesvizhskii et al., 2003) as incorporated by Scaffold including a minimum of four spectral counts per protein. Spectral counts were used as a measure of protein abundance.

### Protein-Protein Interaction (PPI) Network Analysis and Functional Enrichment Analysis

A protein–protein interaction network of the proteins identified with a minimum of four spectral counts in at least three out of five biological replicates was constructed using STRING (v. 11.0, medium confidence of minimum required interaction score = 0.40) (Szklarczyk et al., 2019) and visualized with Cytoscape (version 3.7.1) (Shannon et al., 2003; Doncheva et al., 2019). Proteins were manually assigned to functional groups using the Uniprot database (Apweiler et al., 2004). For functional annotation and enrichment calculation, Database for annotation, visualization and integrated discovery (DAVID), version 6.8 (Huang et al., 2009a; 2009b) was used by utilizing DAVID_CC subontology list (Benjamini–Hochberg corrected *p*-value < 0.05, EASE score < 0.1, at least four identified proteins). To visualize the enrichment, REViGO tool was used (with allowed similarity: small (0.5), semantic similarity measure to use: Resnik-normalized) (Supek et al., 2011).

### Transmission Electron Microscopy

Cal27 and 2B1 cells were grown on Aclar film (Agar Scientific Ltd.) for 7 days in culture medium and fixed with 4% (v/v) formaldehyde plus 2.5% (v/v) glutaraldehyde in 0.1 M HEPES buffer (pH 7.2). Subsequently samples were post-fixed with 1% (w/v) osmium tetroxide and 1.5% (w/v) potassium ferricyanide in 0.1 M cacodylate buffer (pH 7.2) for 1 h, then 1% (w/v) tannic acid in 0.1 M cacodylate buffer (pH 7.2) for 1 h and finally in 1% (w/v) uranyl acetate in distilled water for 1 h. Samples were then dehydrated in an ethanol series infiltrated with TAAB Low Viscosity resin and polymerized for 24 h at 60°C as thin layers on Alcar sheets. After polymerization Aclar sheets were peeled off and layers of polymerized resin with cells were re-embedded with the same resin as stacks. Sections were cut with a Reichert Ultracut ultramicrotome and observed with a FEI Tecnai 12 Biotwin microscope at 100kV accelerating voltage. Images were taken with a Gatan Orius SC1000 CCD camera.

### SDS-PAGE and Western Blot

Total cell lysates were obtained by lysing 1.2 × 10^6^ cells in 200 μl RIPA buffer (Thermo Fisher Scientific, United States), mixed with 5 × Laemmli loading buffer (125 mM Tris-HCl (pH 6.8), 25% (w/v) glycerol, 10% (w/v) SDS, 0.01% (w/v) bromophenol blue, 20% (v/v) 2-mercaptoethanol) to reach a final 1× concentration and heated for 5 min at 96°C. Isolated IACs from at least six 10 cm diameter culture dishes were dissolved in 2× Laemmli loading buffer and heated for 20 min at 70°C. Total cell lysates or isolated IACs were analyzed by SDS-PAGE and WB. Isolated IACs were loaded onto gradient pre-cast gels (Biorad, United States), separated with SDS-PAGE and transferred to a nitrocellulose membrane (Amersham, Germany). The membrane was blocked in 5% (w/v) non-fat dry milk, and incubated with the appropriate antibodies, followed by incubation with horseradish peroxidase coupled secondary antibody. All antibodies are listed in Supplementary Table S1. Detection was performed using chemiluminescence (GE Healthcare) and visualized using iBright CL1000 (Thermo Fisher Scientific, United States) or Uvitec Alliance Q9 mini (BioSPX b.v., Netherlands).

### Statistical Analysis

MTT experiments were repeated at least three times, expressed as mean ± standard deviation (SD) and analyzed by related-measure two-way analysis of variance (ANOVA) with Bonferroni posttest in GraphPad Prism v8.0 (GraphPad Software, United States) to assess significance. QSpec Spectral counter tool was used for MS data to measure the significance of differentially identified proteins in 2B1 versus Cal27 (Choi et al., 2008, 2015) and GraphPad Prism v. 8 was used for visualization.

## Results and Discussion

### Cal27 Cells Primarily Utilize Integrin α6β4 for Adhesion

To define the adhesome composition in Cal27 and Cal27-derived 2B1 cells with *de novo* expression of integrin αVβ3 (Stojanović et al., 2016) we first optimized the IAC isolation and defined the adhesome for the Cal27 cell line. IACs were isolated from cells in long-term culture (72 h), without prior coating of the cell culture dish with ECM proteins (Jones et al., 2015). This approach was selected because it enables the analysis of IAC proteins as well as cell-secreted ECM proteins, as shown recently (Paradžik et al., 2020). The optimal crosslinking duration of 10 min was selected based on WB analysis of the marker IAC components, focal adhesion kinase (FAK), talin 1 (TLN1), integrin linked kinase (ILK) and paxillin (Horton et al., 2015) from the isolated IACs (Supplementary Figure S1).

**FIGURE 1:**
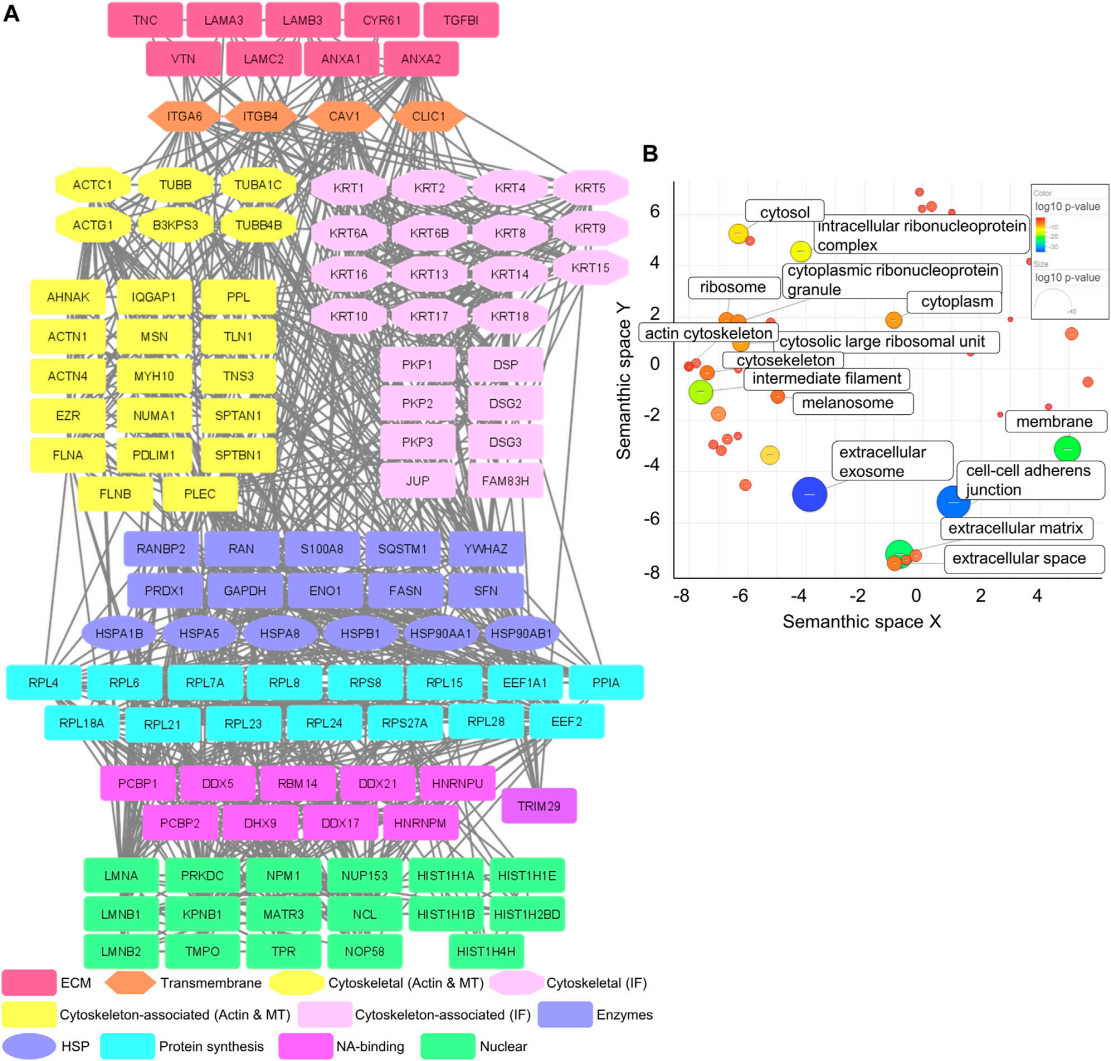
Mass spectrometry analysis of IACs isolated from Cal27 cells. **(A)** Protein–protein interaction network of proteins identified in IACs isolated from Cal27 cells. Shapes represent proteins identified via MS and are labelled with gene symbols and manually grouped and assigned color according to their functional group, as indicated on the left. Only proteins identified with a minimal number of four spectral counts in at least three out of five analyzed biological replicates, FDR < 5%, probability for protein identification ≥ 99.9% were visualized. The network was generated with Cytoscape 3.7.2. **(B)** IACs isolated from Cal27 were enriched with proteins connected to the membrane, cytoskeleton and ECM components. Proteins from **(A)** were assigned to functional groups using the DAVID GO database (GOTERM_CC_DIRECT) and visualized using the REViGO tool, where *p*-values related to GO terms of cellular components were represented by the color bar and size of the circle. Statistically significant GO terms (*p* > 0.05) are presented.

MS analysis of the Cal27 IACs detected 120 proteins with at least four spectral counts in minimum three out of five analyzed replicates (Supplementary Table S2.1), 68 of which were in the meta adhesome (Horton et al., 2015). The only integrin subunits identified were α6 (ITGA6) and β4 (ITGB4), indicating that Cal27 in cell culture primarily utilize α6β4 for adhesion. Classically, α6β4 forms HDs, which are multiprotein complexes that facilitate the stable adhesion of basal epithelial cells to the underlying BM. The extracellular region of α6β4 binds to laminin ligands, in particular the epithelial BM-specific variant laminin-332 (Aumailley et al., 2005; Walko et al., 2015). Consistent with this, the Cal27 IAC preparations contained all three chains of laminin-332 (laminin subunit α3 (LAMA3); laminin subunit β3 (LAMB3) and laminin subunit γ2 (LAMC2)) (Figure 1A).

To further analyze the dataset, gene ontology (GO) enrichment analysis was performed on proteins identified in IACs isolated from Cal27 cells. A significant enrichment of GO terms related to extracellular exosome, cell-cell adherens junctions, ECM, membrane, intermediate filament and intracellular ribonucleoprotein complex was observed. Other GO terms such as ribosome and actin cytoskeleton were less well represented (Figure 1B and Supplementary Table S2.2). Therefore, the GO analysis supports the successful isolation of IACs from Cal27 cells.

Our previously published data showed that Cal27 cells express integrin β1, β5, β6 and αV mRNAs, cell surface αVβ5, and adhere to vitronectin (VTN) and FN (Stojanović et al., 2016). Therefore, we expected that the composition of ECM proteins secreted by Cal27 cells reflects this integrin profile. We identified several ECM components, including established integrin ligands such as VTN, tenascin C (TNC) and cysteine-rich 61 (CYR61) (Figure 1A). VTN is recognized by αVβ1, αVβ3, αVβ5 and αIIbβ3 (Felding-Habermann and Cheresh, 1993), TNC by αVβ1, αVβ3, αVβ6, α2β1, α9β6 and α8β1 (Midwood et al., 2016) and CYR61 by αVβ3 and αVβ5 (Lau, 2016). We also identified annexins A1 and 2 (ANXA1/2), for which both cellular and extracellular localizations have been demonstrated (Wang and Lin, 2014; Boudhraa et al., 2016), and transforming growth factor beta induced (TGFBI) which connects various ECM components, and interacts with several integrins including α1β1, α3β1, αVβ3, and αVβ5 (Ween et al., 2012). We also detected caveolin-1 (CAV1), a structural protein of caveolae/lipid rafts involved in integrin-dependent signaling (Echarri and Del Pozo, 2006), and chloride intracellular channel 1 (CLIC1), upregulated in several cancers (Peretti et al., 2015).

Our recently published data, obtained by differential analysis between melanoma cells MDA-MB-435S and MDA-MB-435S-derived cell clones with decreased expression of integrin αV, identified key components of integrin αVβ5 adhesion complexes, namely talin 1 and 2 (TLN1 and 2), α-actinin 1 and 4 (ACTN1 and 4), filamin A and B (FLNA and B), PLEC and vinculin (VCL) (Paradžik et al., 2020), which are all part of the consensus adhesome (Horton et al., 2015). All these proteins, except TLN2, were detected in Cal27 IACs, whereas VCL was detected in only two out of five samples. These data indicate that Cal27 cells in long term culture do form FAs, despite the fact that the relevant integrin subunits were not detected by MS (Figure 1A). Therefore, these data suggest that αV integrin heterodimers are likely to be a low abundant component of the Cal27 adhesome under the conditions tested.

Type I HDs, found in stratified and pseudostratified epithelia, consist of integrin α6β4, PLEC, CD151, BP230 and BP180 (Owaribe et al., 1990). Type II HDs, found in simple epithelia, consist only of integrin α6β4 and PLEC (Uematsu et al., 1994; Fontao et al., 1999). Since the main integrin identified in IACs from Cal27 cells was α6β4, we searched the MS data for other HD components. Out of the known HD-specific proteins, we detected only PLEC suggesting that Cal27 cells form type II HDs.

Type II HDs are characterized by electron-dense regions connected to cytokeratin filaments (Fontao et al., 1997). It is well known that intermediate filaments assembled from basal cell keratins KRT-5 and KRT-14 associate with the inner plaque of HDs via PLEC and BP230 (Walko et al., 2015). Interestingly, both KRT-5 and KRT-14 and a plethora of additional keratins, i.e., KRT-1/2/4/6A/6B/8/9/10/13/15/16/17/18 were identified in Cal27 IACs. Since keratins can originate from MS sample preparation contamination (Hodge et al., 2013) and provide false positives, we evaluated their significance by comparing the number of spectra for these keratins in Cal27 isolates with the adhesome of human breast carcinoma MDA-MB-231 cells which were simultaneously analyzed (unpublished data, data not shown). The most important difference was in the number of spectra for KRT-5 and KRT-14, which were represented with the highest number of spectra of all keratins and had a 10-fold higher number of spectra in Cal27 cells as compared to MDA-MB-231 cells (unpublished data, data not shown). In addition, a striking difference was observed in KRT-15/16/17 which were absent in IACs from MDA-MB-231 cells but present in Cal27 cells (Supplementary Table S2.1). Indeed, it has been demonstrated that the expression of KRT-17 is higher in oral squamous cell carcinoma tissues compared to non-tumor tissues (Coelho et al., 2015) but is also expressed in breast carcinoma (Turashvili et al., 2007). Conversely, KRT-19 was not detected in the Cal27 adhesome unlike the MDA-MB-231 adhesome (Supplementary Table S2.1). Together, these data indicate that enrichment in KRT-5 and KRT-14 is a feature of Cal27 cells, which further supports the formation of HDs by integrin α6β4. However, these data do not allow us to conclude whether KRT-5 and KRT-14 are an integral part of HDs. Therefore, based on the adhesome data we hypothesize that Cal27 cells in cell culture adhere preferentially through formation of type II HDs.

A constructed PPI network (Figure 1A) further shows a group of desmosomal proteins, i.e., periplakin (PPL), desmogleins 2 and 3 (DSG2/3), plakophilins 1, 2 and 3 (PKP1/2/3), junction plakoglobin (PLAK) and desmoplakin (DSP). These proteins are not frequently detected in adhesome analysis and were absent in the MDA-MB-435S adhesome analyzed in the same manner (Paradžik et al., 2020). However, melanoma cells MDA-MB-435S are metastatic and express mesenchymal markers, such as vimentin (Han et al., 2014), unlike Cal27 cells which possess an epithelial phenotype (Yadav et al., 2011). DSGs and PLAK, but also integrins α6 and β4, were detected in two types of human oral squamous carcinoma cell lines by MS in deroofed cells attached to glass coverslips (Todorović et al., 2010).

MS analysis identified heat shock proteins and many ribosomal proteins (ribosomal protein large (RPL) and small (RPS)) and those related to RNA and protein synthesis such as DEAD box proteins (DDX). A plausible explanation is that adapter proteins, which interact with integrin mRNAs, could support specific integrin dimer formation (Hatzfeld and Magin, 2019). Actually, ribosomes have been found to co-localize with β3 integrin-enriched FAs on engagement with ECM proteins (Willett et al., 2010). Similarly, the α6 integrin 3′UTR is essential not only for the formation and localization of the α6β4 heterodimer to cell-matrix adhesions but also its stability (Woychek et al., 2019). Finally, the Cal27 PPI network contains a group of nuclear proteins (Figure 1A).

In conclusion, our results indicate that Cal27 cells use preferentially, but not exclusively, integrin α6β4 for adhesion in cell culture, unlike melanoma MDA-MB-435S (Paradžik et al., 2020), osteosarcoma U2OS, lung carcinoma A549 and melanoma A375 (Lock et al., 2018) which all use integrin αVβ5. The composition of the Cal27 adhesome and the absence of BP180 and BP230 indicate that these cells form type II HDs composed of α6β4 and PLEC, but these data do not allow us to conclude whether KRT-5 and KRT-14, which represent the majority of keratins in this cell line, are linked to these HDs.

### Comparison of Cal27 and 2B1 Adhesomes Suggests That *De Novo* Expression of Integrin αVβ3 Increased the Abundance of FAs and Type II HDs

**FIGURE 2:**
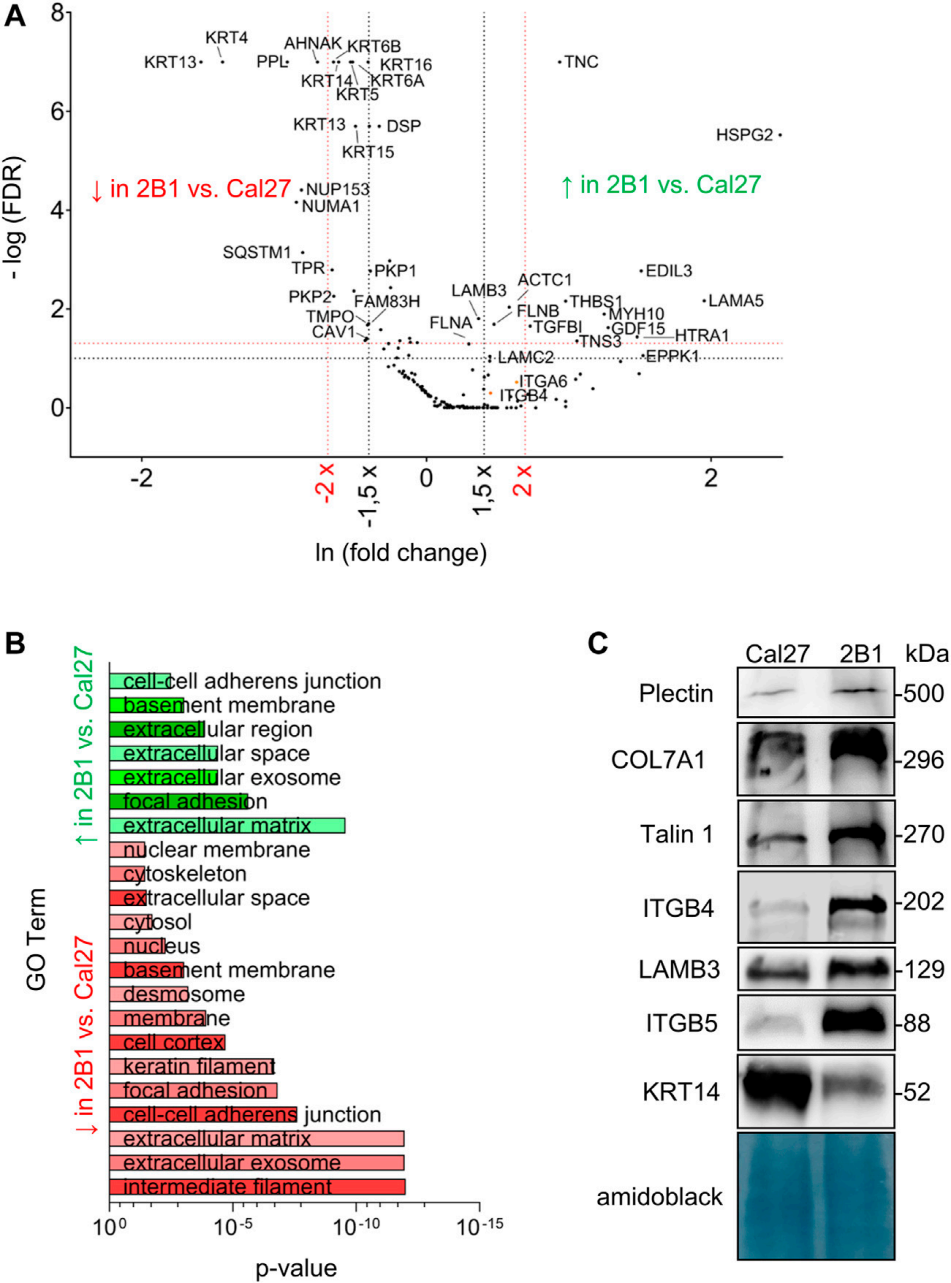
Analysis and validation of IACs isolated from Cal27 cells and clone 2B1. **(A)** Volcano plot analysis of proteins detected in IACs isolated from Cal27 cells versus clone 2B1. IAC proteins from Cal27 and 2B1 cells are visualized as volcano plot after the analysis with QSpec/QProt to generate -Log (FDR) and fold change values. Cut off values of −Log (FDR) ≥ 1 (red horizontal dotted line) corresponding to FDR ≤ 0.05 and −Log (FDR) ≥ 1,3 (black horizontal dotted line) corresponding to FDR ≤ 0.1; and fold change ≥ 1.5 (black vertical dotted line) or 2 (red vertical dotted line) were used. Each dot on the plot represents 1 protein. Proteins with significantly different abundance between IACs of Cal27 and 2B1 cells, and of interest for this paper are marked with their gene name. Upper left quadrant–proteins detected with lower levels of spectra in 2B1, upper right quadrant–proteins detected with higher levels of spectra in 2B1, compared to Cal27. For this analysis, only proteins identified with a minimal number of spectral counts ≥ 4 in at least three out of five biological replicates in either of Cal27 or 2B1 set were used, FDR < 5%, probability for protein identification ≥ 99.9% were visualized. **(B)** DAVID GO analysis of proteins from **(A)** with FDR ≤ 0.1 and fold change ≥ or ≤ 1.5, detected with higher (green) and lower (red) abundances in 2B1 as compared to Cal27. Statistically significant GO terms were presented in reverse *x*-axis of *p*-value from lowest (top) to the highest significance (bottom). Green represents GO terms annotated to the proteins whose abundance is higher in 2B1 than Cal27, and red to reverse. The *p*-value represents Benjamini corrected *p*-value. **(C)** WB analysis of IAC proteins from Cal27 and 2B1 cells. Seventy-two hours after seeding, IACs were isolated and WB analysis was performed. The results presented are representative of two independent experiments yielding similar results. PLEC, plectin; COL7A1, collagen VII; TLN1, talin 1; ITGB4, integrin subunit β4; LAMB3, laminin subunit β3; ITGB5, integrin subunit β5; KRT-14, keratin 14.

Since our previous data showed *de novo* expression of integrin αVβ3 in Cal27 cells induced differences in sensitivity to anticancer drugs (Stojanović et al., 2016), we aimed to identify IAC proteins that contributed to the observed phenotype changes (Supplementary Table S2.3). MS identified differences in IAC composition between Cal27 and 2B1 cells (Figures 2A, B). Since 2B1 cells have increased expression of both αVβ3 and αVβ5, and demonstrated increased adhesion to both FN and VN (Stojanović et al., 2016), we expected to detect more FA proteins in 2B1 compared to Cal27 cells. Indeed, we observed higher levels of proteins implicated in FA formation, i.e., FLNB, tensin-3 (TENS3) and myosin 10 (MYH10) in the 2B1 adhesome. FLNB and TENS3 were both found to be part of the integrin αVβ5 adhesome (Paradžik et al., 2020). FLNB is an actin-binding protein whose expression has been associated with invasiveness in osteosarcoma and radioresistant lung cancer cells (Iguchi et al., 2015), in line with higher expressing and more invasive 2B1 cells. Tensins are regulators of Rho GTPase signaling and cell adhesion (Blangy, 2017). An unbiased assessment of IAC proteins with higher abundance in 2B1 compared to Cal27 cells using DAVID GO analysis suggested that they are mostly components of the ECM and FAs. Conversely, proteins present in lower levels in clones 2B1 compared to Cal27 cells were classified as intermediate filaments, ECM and cell-cell adherent junctions (Figure 2B and Supplementary Table 2.3).

Other proteins detected at higher levels in IACs from 2B1 compared to Cal27 were the ECM proteins TNC, thrombospondin-1 (THBS1), TGFBI and EGF-like repeat, discoidin I-like domain-containing protein 3 (EDIL-3), high-temperature requirement serine protease (HTRA1) and heparan sulphate proteoglycan 2 (HSPG2, perlecan). Although the spectral counts for THBS1, EDIL-3, HTRA1 and HSPG2 in Cal27 cells were below the selected cut off value (Supplementary Table S2.3), they showed increased abundance in 2B1 cells. TNC is an ECM protein which binds to many different integrin heterodimers, especially αVβ3, but not αVβ5 (Midwood et al., 2016) and THBS1 is an extracellular mediator of matrix mechanotransduction that acts via integrin αVβ1 to establish FAs (Yamashiro et al., 2020). Therefore, their increased abundance in 2B1 IACs very likely reflects the *de novo* expression of integrin αVβ3-containing FAs in 2B1 cells and/or increased expression of integrin αVβ5 (Stojanović et al., 2016). THBS1 is also closely associated with transforming growth factor β (TGF-β). Latent TGF-β can be activated through different mechanisms, including integrins in concert with mechanical forces due to ECM stiffness and/or cytoskeletal forces, or by binding to the secreted and ECM protein THBS1 (Atanasova et al., 2019). TGFBI, an ECM interacting protein, activates the FAK signaling pathway through its binding to integrin αVβ5, enhances glycolysis and promotes pancreatic cancer cell migration (Costanza et al., 2019) which is in line with our data of increased migration of 2B1 cells. The observed higher levels of actin are also in line with migration data (Stojanović et al., 2016). Higher level of EDIL3 in 2B1 cells is consistent with the increased amount of integrin αVβ3 FAs (Stojanović et al., 2016). EDIL-3 (DEL-1) is a ECM protein that promotes adhesion of endothelial cells through interaction with the αVβ3 (Hidai et al., 1998; Yuh et al., 2020). The connection between integrin αVβ3 and EDIL3 has been shown in HNSCC samples compared with non-cancerous controls. Namely, Cen et al. (2018) demonstrated decreased expression of MiR-375-3p, thus acting as a tumor suppressor via regulating tumor-related genes LAMC1, EDIL3, FN1, VEGFA, IGF2BP2, and IGF2BP3 in HNSCC, which were greatly enriched in the pathways of integrin β3 cell surface interactions. Moreover, EDIL3 is significantly correlated with mesenchymal phenotype, angiogenesis, and tumor progression in lung adenocarcinoma (Jeong et al., 2017). It also promotes epithelial–mesenchymal transition (EMT) and paclitaxel resistance through interaction with integrin αVβ3 in cancer cells and its blockade by cilengitide restores sensitivity and reverts EMT (Gasca et al., 2020). HTRA1, a serine protease shows higher levels in 2B1 IACs compared to Cal27. It has a variety of targets, including ECM proteins such as FN (Jiang et al., 2012). Higher levels of HSPG2 (perlecan) in 2B1 is consistent with data showing that intercellular deposition of HSPG2, a basement-membrane type heparan sulphate proteoglycan which can interact with β1 and β3 integrins (Hayashi et al., 1992), is enhanced in oral epithelial dysplasia and carcinoma *in situ* (Hasegawa et al., 2016). In conclusion, many proteins detected at higher levels in IACs from 2B1 compared to Cal27 are related to increased amount of integrins, either αVβ3 and/or αVβ5, detected previously (Stojanović et al., 2016).

As we emphasized previously, the main integrin used by Cal27 cells in long term culture is integrin α6β4. Interestingly, we found increased levels of both integrin subunits α6 and β4 in IACs of 2B1 cells, compared to Cal27, although this difference wasn’t statistically significant. We wondered whether laminin-332, the main laminin used by integrin α6β4 for adhesion (Walko et al., 2015), was also present in higher amounts in 2B1 cells. The IAC MS data showed higher levels of all three laminin subunits LAMA3, LAMB3 and LAMC2 (only LAMA3 difference wasn’t statistically significant) in IACs from 2B1 cells compared to Cal27 (Supplementary Table S2.3), indicating that 2B1 cells secrete an increased abundance of laminin-332. Interestingly, higher levels of the α6β4 interacting ECM protein laminin subunit α5 (LAMA5) were also detected in 2B1. LAMA5 is a part of laminin-511 (α5β1γ1), a potent adhesive and pro-migratory ECM substrate for a variety of normal and tumor cell lines *in vitro* (Pouliot and Kusuma, 2013). In Cal27 neither laminin β1 nor γ1 specific spectra were observed, while in 2B1 none or very low number of spectra in different samples for laminin β1 or γ1 were detected, thus preventing us to conclude on the actual difference in laminin-511 expression between the two cell lines (Supplementary Table S2.3).

Many proteins were found to be present in lower amounts in IACs from 2B1 compared to Cal27 (Figure 2A; Supplementary Table S2.3) including many keratins, i.e., KRT-5, -6A, -6B, -13, -14, -15, -16 and -17. KRT-5 and KRT-14 are the main keratins employed by Cal27 cells. This finding was surprising, as 2B1 cells showed an increased amount of integrin α6 and β4 subunits. This indicated that they are not part of type II HDs. Te Molder et al. (2019) described hybrid cell-matrix adhesions which are present in the central region of the cells containing CD151 - α3β1/α6β4 integrin complexes and PLEC, which were not anchored to the keratin filaments. In addition, CD151 was necessary for proper organization of these integrins in the central region of the cells. However, we did not observe CD151 nor integrin α3 in the adhesome of Cal27 or 2B1 cells.

Other proteins found at lower levels in IACs from 2B1 cells were PPL and DSP. PPL is a member of plakin family of proteins implicated in crosstalk between three major cytoskeletal networks. PPL, together with DSP, envoplakin, and epiplakin is predominantly involved in intermediate filament binding as components of desmosomes and the cornified envelope (Quick, 2018). Since PPL is mostly downregulated in cancer, e.g., in esophageal squamous cell carcinoma (Nishimori et al., 2006) and many other cancers regulating cancer cell growth, survival, migration, invasion and drug resistance (Wesley et al., 2021), its downregulation in more therapy resistant and more migratory 2B1 cells (Stojanović et al., 2016) was expected. In Cal27 cells we detected almost all components of desmosomes, i.e., DSG 2/3, PKP 1/2/3, PLAK and DSP. We also detected KRT-5 and KRT-14 filaments which were shown to support stable desmosomes (Loschke et al., 2016). Both plakophilins (PKP1/2), DSP, KRT-5 and KRT-14 were less abundant in 2B1 cells while DSG 2/3 were detected with low number of spectra preventing us to conclude on its differential expression. These data indicate that both cell lines form desmosomes which are less abundant in 2B1 compared to Cal27 cells, and indicate that KRT-5 and KRT-14 filaments are very likely anchored with desmosomes. The observed reduced cell-cell contact in 2B1 compared to Cal27 cells is in line with increased migration of 2B1 compared to Cal27 (Stojanović et al., 2016). It remains to be determined how desmosome components are retained through the IAC isolation protocol.

Additional proteins found in lower amount in 2B1 cells compared to Cal27 were two nuclear proteins, nuclear mitotic apparatus protein 1 (NUMA1) and nucleoporin 153 (NUP153). Finally, neuroblast differentiation-associated protein AHNAK is also present in reduced amount in 2B1 IACs. AHNAK was found in several adhesomes (Kuo et al., 2012; Horton et al., 2015; Paradžik et al., 2020), in RAs (Lock et al., 2018) and as β4 interacting protein (Myllymäki et al., 2019; Wang et al., 2020) and was shown to function as a tumor suppressor via modulation of TGFβ/Smad signaling pathway (Lee et al., 2014). However, its involvement in tumor progression is still unknown.

### Validation of IAC Proteins Differentially Detected in Cal27 and 2B1 Adhesomes

The increased abundance of β4 in IACs of 2B1 cells was confirmed using WB of isolated IACs (Figure 2C), while increased expression of integrin subunit α6 in 2B1 was confirmed by flow cytometry (Supplementary Figure S2A). The formation of HDs can be initiated through laminin-332 deposited by the cells (Litjens et al., 2006). Therefore, the increased level of HDs is in line with increased deposition of corresponding ECM proteins, i.e., laminin-332 whose increased expression in 2B1 was confirmed by WB of LAMB3 (Figure 2C) supporting the MS analysis of all laminin-332 components in IAC isolates. We have also found COL7A1 expression in IAC isolates of both Cal27 and 2B1 cells and the expression was increased in 2B1 (Figure 2C). Type VII collagen (COL7A1) is a major component of anchoring fibrils, providing mechanical strength via linking the basal lamina and the underlying connective tissue, and is synthesized by keratinocytes and fibroblasts (Goletz et al., 2017). The cytoskeletal linker PLEC, part of HDs type I and II, was shown to mediate crosstalk between HDs which oppose force transduction and traction force generation by FAs by coupling intermediate filaments to the actin cytoskeleton (Zuidema et al., 2020). In accordance, WB analysis of IAC isolates demonstrated increased levels of PLEC in 2B1 cells compared to Cal27 (Figure 2C) in contrast to the MS analysis which indicated similar amounts (Supplementary Table S2.3). Although the classical type I HD component BP180 was not detected by MS, we did observe similar BP180 expression in both cell lines by WB and the BP180 was weak, if at all present, in isolated Cal27 and 2B1 IACs (data not shown). These results support the conclusion that both cell types form type II HDs. Finally, we confirmed decreased levels of KRT-14 in 2B1 cells compared to Cal27 (Figure 2C), thus supporting the conclusion that HDs in Cal27 and 2B1 cells have reduced anchorage to KRT-5 and KRT-14. Interestingly, WB analysis of KRT-14 in Cal27 and 2B1 cells upon integrin β4 knockdown showed that the expression of KRT-14 does not change in Cal27 or 2B1 cells upon integrin β4 knockdown (Supplementary Figure S2B).

Integrin subunit β5 was not detected by MS in Cal27, but in 2B1 cells there was a low detection in 1 repeat, while integrin subunits αV and β3 were not detected at all. However, the expression of integrin heterodimer αVβ5 in Cal27 and its increased expression in 2B1 cells, as well as the *de novo* expression of αVβ3 in 2B1 cells was demonstrated previously (Stojanović et al., 2016). In addition, the difference in αVβ5 integrin abundance in Cal27 and 2B1 IAC isolates was confirmed using WB (Figure 2C). Although TLN1 was found at low levels by MS in IACs, WB analysis demonstrated increased levels of TLN1 in 2B1 cells compared to Cal27, demonstrating further increased amount of FAs in 2B1 compared to Cal27 (Figure 2C).

Analysis of the Cal27 and 2B1 adhesome upon long-term culture detected only α6 and β4 integrin subunits, indicating that these cells preferentially, but not exclusively, use integrin α6β4 for adhesion. We have recently published results of adhesome analysis of human melanoma cell line MDA-MB-435S in which we found predominantly integrin subunits αV and β5 (Paradžik et al., 2020), that is in accordance with our unpublished results for human melanoma cell line RPMI-7951 and breast carcinoma cell line MDA-MB-231, as well as with results published by Lock et al. (2018) in human osteosarcoma U2OS, lung carcinoma A549 and melanoma A375 cells. Unlike Cal27, all these cell lines are highly metastatic cells with more mesenchymal than epithelial characteristics (Yadav et al., 2011). We have shown that 2B1 cells express increased amount of integrin α6β4 on the cell surface. Integrin α6β4 has been shown to form HDs (Walko et al., 2015) and therefore we searched for more HD components and found only PLEC which was more abundant in 2B1 compared to Cal27 cells only in further validation experiments using WB. The differential expression of α6β4 also has an effect on ECM protein characteristics for HDs, i.e., in 2B1 cells we found increased laminin-332 as well as COL7A1, confirming existence of HDs in both cell lines. Interestingly, we also observed increased amount of another BM component, perlecan which is also more abundant in 2B1 compared to Cal27. An important gene expression signature highly expressed in a subset of recurrent HNSCC includes laminin α3, β3 and γ2, components of the laminin-332, as well as integrins α6 and β4 forming integrin α6β4, which serves as its ligand, suggesting a potentially aggressive phenotype prone to invasion and metastasis (Ginos et al., 2004). HNSCC tumor biology is strongly associated with deregulations within the ECM compartment. Laminin-332 is one of the main isoforms associated with malignant transformation, contributing to proliferation, adhesion, migration, invasion, and metastasis (Ramovs et al., 2017), due to its involvement in the regulation of several pathways, including the activation of the EGFR/MAPK as well as PI3K/AKT. Therefore, laminin-332 may represent an attractive potential therapeutic target for these tumors (Meireles Da Costa et al., 2021).

Todorović et al. (2010) used a method for IAC enrichment (deroofing the cells with ammonium hydroxide and the removal of cytosolic and organellar proteins by stringent water wash) and MS analysis of proteins associated with the basal surface of the cell and its underlying ECM. They analysed differential expression in PLAK-null cells compared to PLAK heterozygous mouse keratinocytes and observed strong downregulation of FN, TNC, integrins α6 and β4 and hemidesmosome component BP180. They applied the same method to compare human oral squamous carcinoma lines CAL33 and UM-SCC-1 which originate from tongue and the roof of the mouth, respectively, and again found differential expression of cell-cell adhesion proteins DSG2 and PLAK and integrin α4, thus supporting the important role of desmosomes and HDs in HNSCC. These results indicate regulation of HDs by desmosomes. However, little is known about the regulation of desmosome adhesion by cell-ECM interaction, FAs and HDs. Latest data clearly showed that HDs and FAs affect each other’s distribution. In normal human epithelial keratinocytes they are identified as separate but linked entities which cooperate to coordinate the dynamic interplay between the keratin and actin cytoskeleton (Pora et al., 2019). FAs and HDs share only one component, namely PLEC, while HDs and desmosomes share keratins. Our data indicate that there is crosstalk between FAs, HDs and desmosomes. A better understanding of FAs, HDs and desmosomes will provide novel clues into the molecular mechanisms that regulate adhesion, migration and survival in cancer cells. These studies may reveal new strategies for cancer treatment. In conclusion, our data indicate that *de novo* expression of integrin αVβ3 in Cal27 results in upregulation of αVβ5, either of them contributing to the upregulation of HDs leading to an altered ventral membrane environment with different links from integrins and the ECM to IAC components and the cytoskeleton. MS data suggest that it is possible that a reduced amount of desmosomes has occurred but this should be verified by other methods and is outside the scope of the research presented in this paper.

### Cal27 and 2B1 Cells Form Type II HD-Like Structures

**FIGURE 3:**
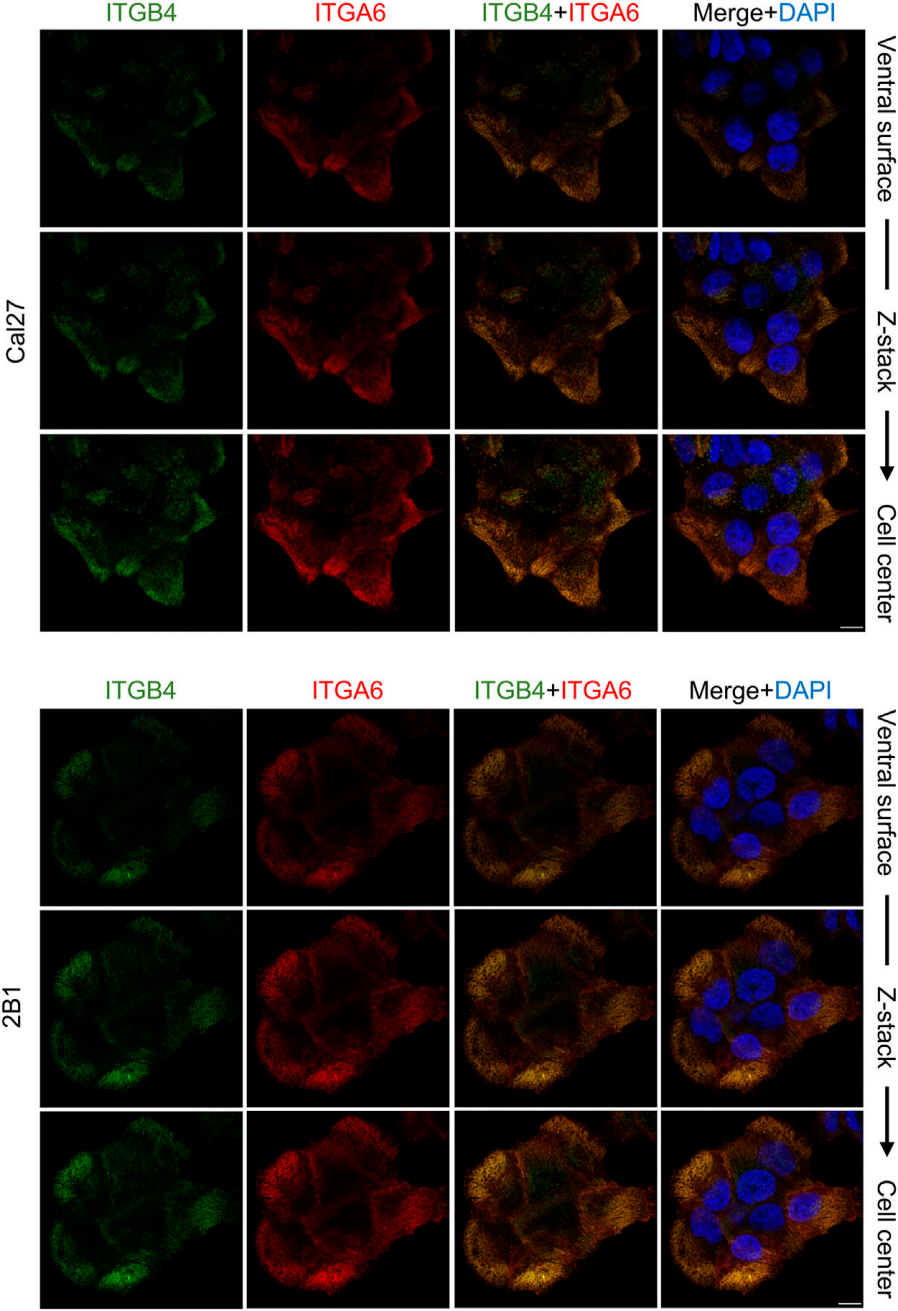
Integrin subunits α6 and β4 co-localize in both Cal27 or 2B1 cells. Confocal z stack images of Cal27 and 2B1 cells. Forty-eight hours after seeding on coverslips, cells were fixed, permeabilized, incubated with antibodies against integrin anti-β4 (ITGB4) antibody followed by Alexa-Fluor 488-conjugated antibody (green) and integrin anti-α6 (ITGA6) antibody followed by Alexa-Fluor 555-conjugated antibody (red). Nuclei were stained with DAPI (blue). Analysis was performed using TCS SP8 Leica. Scale bar = 10 μm.

Our MS analysis of the Cal27 cell adhesome identified only type II HD components, α6β4 integrin and PLEC (Supplementary Table 2.1). Differential analysis showed increased levels of integrins α6, β4 and PLEC, and decreased levels of KRT-5 and KRT-14 in IACs from 2B1 cells, suggesting a reduced link of type II HDs adhesions with keratins. To analyze the cellular localization of integrin heterodimer α6β4 we performed IF analysis using antibodies directed against integrins α6 and β4 and demonstrated typical integrin α6 and β4 staining, i.e., diffuse ventral membrane localization in cauliflower or leopard skin pattern (Figure 3). We also performed IF analysis using antibodies against KRT-14 (Supplementary Figure S3). Surprisingly, we observed a different KRT-14 pattern between the cell types. In Cal27 cells KRT-14 was distributed at cell edges, whilst in 2B1 cells KRT-14 was more diffuse, and the KRT-14 network was dispersed throughout the cytoplasm, which was particularly visible when moving away from the bottom of the cell into the cell body.

**FIGURE 4:**
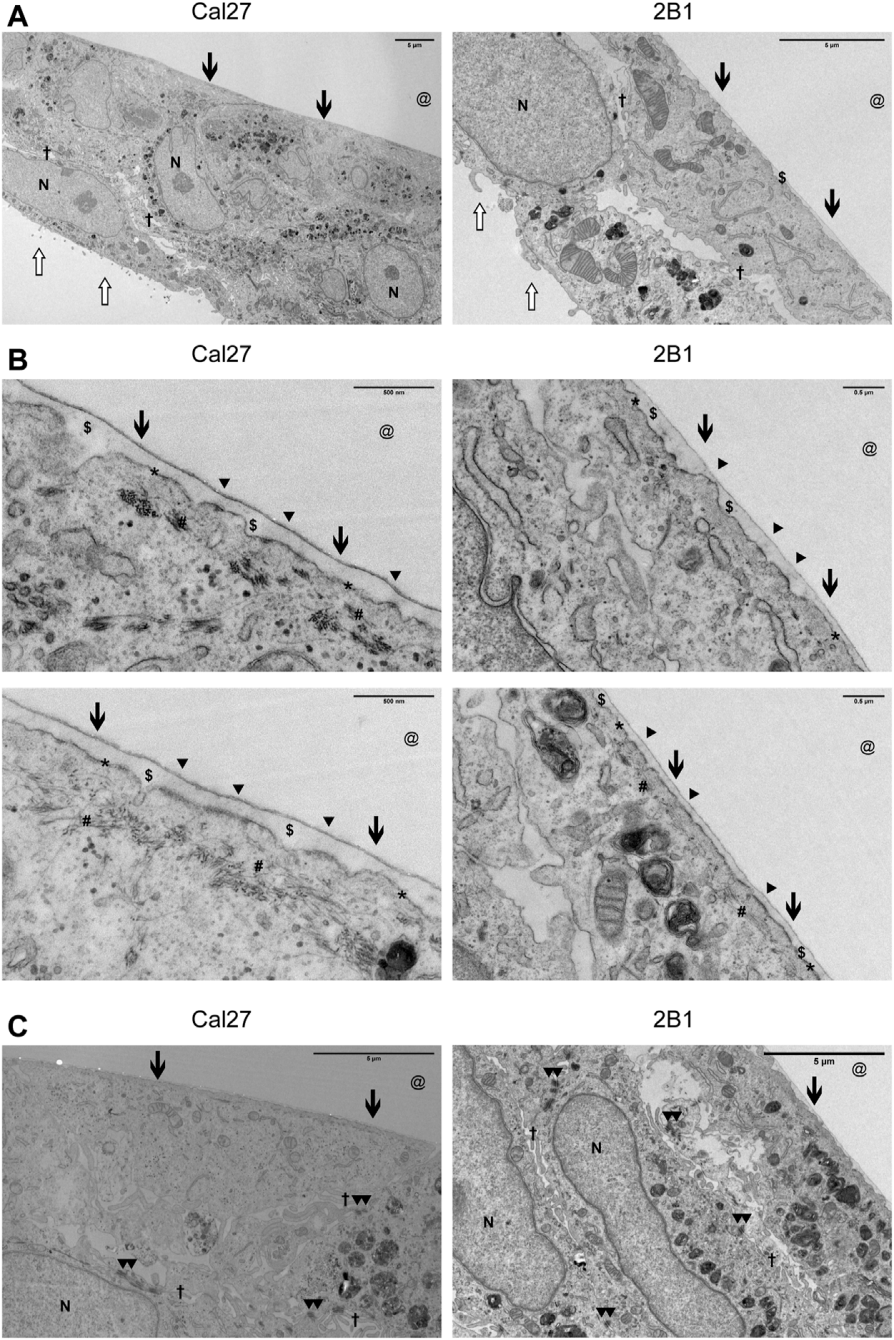
Cal27 and 2B1 cells form HD-like structures which are indicative as type II HDs. **(A–C)** Cal27 and 2B1 cells were cultured on Aclar for up to 7 days and transverse sections of the cell-ECM interface were prepared, imaged by TEM, and a range of magnifications shown. **(A)** Lower magnification images of cell monolayers that have formed a flattened basal surface with a thin layer of ECM ($) proximal to the area where the Aclar film (@) would have occupied. **(B)** Higher magnification images of the cell-ECM interface. Arrowheads (^▲^) indicate the approximate position of some type II HDs (indicated from the extracellular side) which are located at the plasma membrane (*) and link to cytoplasmic cytokeratin filaments (#). **(C)** Areas of cells to illustrate the formation of desmosomes. All images are orientated with the cell-ECM interface towards the top of the images. Other symbols used are [N = nucleus; Open arrow = luminal side with microvilli; Filled arrow = basal surface next to ECM and aclar.; † = cell-cell junction where you can sometimes see electron dense desmosomes (^▲▲^) as shown in **(C)**.

Due to the limited resolution of IF microscopy, we analyzed HDs in Cal27 and 2B1 cells by transmission electron microscopy (TEM). In TEM, HDs appear as tripartite structures consisting of an inner and outer plaque and a sub-basal dense plate. The inner hemidesmosomal plaque is composed of the PLEC and BP230 proteins, which are involved in connecting the HD to the keratin intermediate filament system. The outer plaque contains the hemidesmosomal transmembrane proteins α6β4 and BP180. The integrin α6β4, a receptor for laminin-332 in the epidermal basement membrane, binds to the intermediate filament anchoring protein PLEC (Borradori and Sonnenberg, 1999; Walko et al., 2015). Cal27 and 2B1 cells were cultured for 7 days on Aclar to allow transverse sections to be viewed by TEM (Figure 4). Both Cal27 and 2B1 cells formed cell layers of 1–2 cell depth. Polarization of cells was apparent, with flattened ventral membrane surfaces next to a small layer of secreted ECM and microvilli on the dorsal membrane (Figure 4A). We observed many areas with increased plasma membrane density that were in close proximity to the secreted ECM (Figure 4A). In addition, many areas displayed increased plasma membrane density alone. Therefore, in agreement with the MS data, the TEM analysis identified plasma membrane adhesion structures that did not display the fully mature HD stratification observed for type I HDs that contain all HD components (Uematsu et al., 1994; Fontao et al., 1999), and supports the conclusion that Cal27 and 2B1 cells form type II HDs. Of interest, the TEM analysis also revealed the presence of cell-cell junctions such as desmosomes (Figure 4C), that is in line with MS data containing many desmosomal proteins (Supplementary Table S2.3).

### Knockdown of Integrin β4 Confers Resistance to CDDP, MMC and DOX in Both Cal27 and 2B1 Cells

The *de novo* expression of integrin αVβ3 in Cal27 cells confers resistance to cDDP, MMC and DOX (Stojanović et al., 2016). Since we found increased expression of integrin α6β4 in 2B1 compared to Cal27 cells we aimed to investigate the possible involvement of α6β4 integrin in sensitivity to these anticancer drugs. We transfected Cal27 and 2B1 cells with siRNA specific for integrin β4 and measured sensitivity to anticancer drugs as compared to the cells transfected with control siRNA. WB confirmed the decreased expression of integrin β4 (Supplementary Figure S2B). Of interest, this analysis also revealed that integrin β4 expression was increased in 2B1 total cell lysate compared to Cal27. To test whether integrin β4 knockdown decreased the amount of integrin α6β4 heterodimer at the cell surface we measured the expression of integrin subunit α6 using flow cytometry. Indeed, integrin β4 knockdown decreased the amount of α6 integrin subunit on the cell surface (Supplementary Figure S2A), thus confirming that integrin β4 knockdown decreases the expression of α6β4 heterodimer at the cell surface.

**FIGURE 5:**
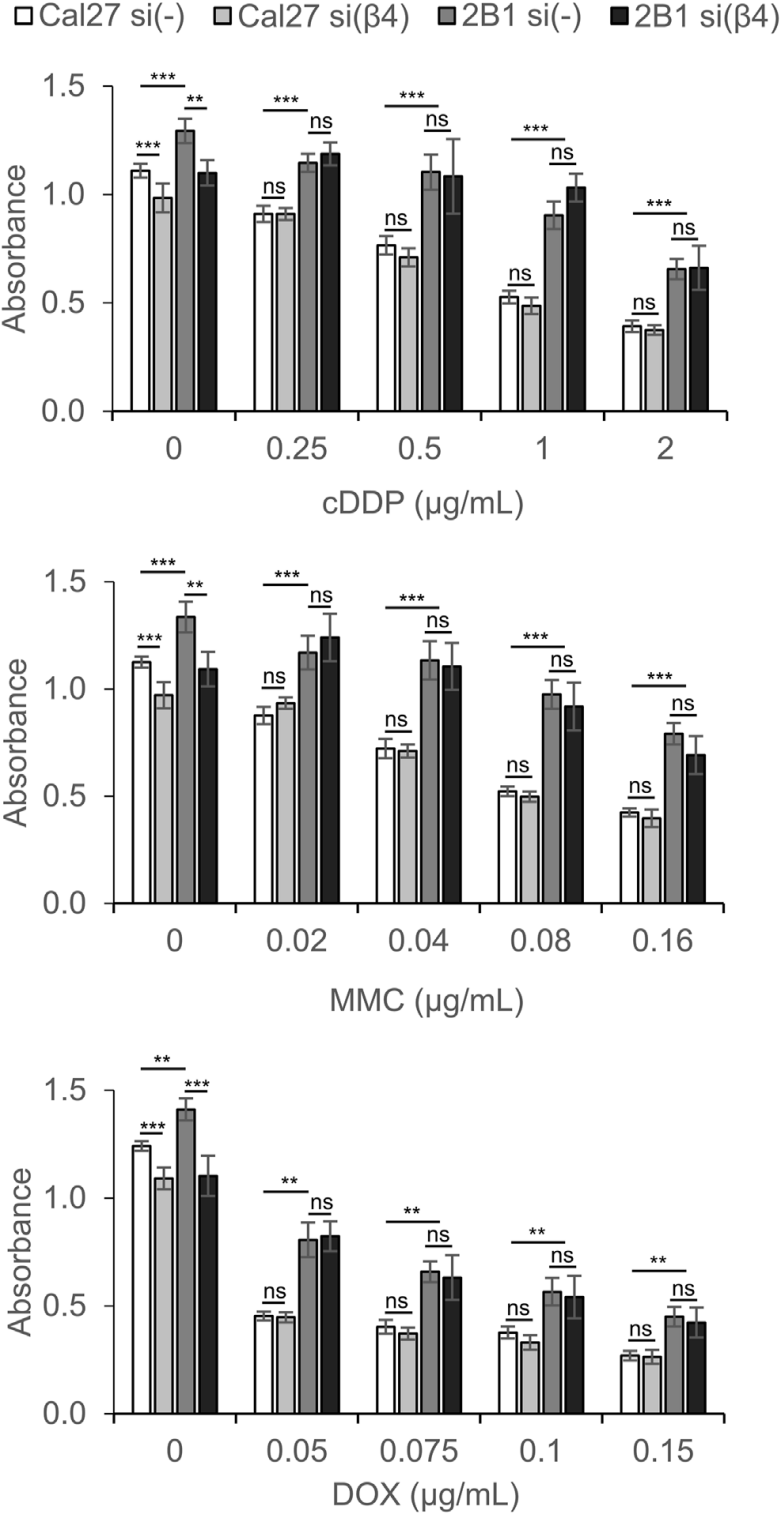
Cal27 and 2B1 cells demonstrate decreased sensitivity to CDDP, MMC or DOX upon knockdown of integrin β4 (si(β4)) as compared to control Cal27 and 2B1 cells transfected with control siRNA (si(-)). Twenty-four hours upon siRNA transfection, cells were seeded in 96-well plates and 24 h later treated with different concentrations of CDDP, MMC or DOX. Cytotoxicity was measured by MTT assay 72 h later. Average absorbance data ± S.D. indicating survival, are representative of at least three independent experiments yielding similar results. Data were analyzed by two-way ANOVA with Bonferroni post-test. ns, not significant; **p < 0.01; ***p < 0.001.

Both Cal27 and cell clone 2B1 transfected with control siRNA retained similar sensitivity to cDDP, MMC and DOX as nontransfected cells (data not shown). However, Cal27 and 2B1 cells transfected with β4-specific siRNA demonstrated resistance, i.e., decreased sensitivity to cDDP, MMC and DOX compared to cells transfected with control siRNA (Figure 5). In the absence of the anticancer drug, both Cal27 and 2B1 cells transfected with integrin β4-specific siRNA demonstrate decreased proliferation compared to control transfection. Upon exposure to different concentrations of cDDP, MMC or DOX, the resistance of 2B1 cells compared to Cal27, both transfected with control siRNA, was retained which corresponds to previously published data (Stojanović et al., 2016). However, in both cells transfected with integrin β4-specific siRNA we observed increased survival (resistance) compared to their own controls. This result confirms the involvement of integrin α6β4 in signaling pathways affecting sensitivity of both cell lines to anticancer drugs (Figure 5). However, since we observed a similar effect of decreased expression of α6β4 in both Cal27 and 2B1 cells, we conclude that mechanisms of anticancer drug resistance triggered by *de novo* expression of integrin αVβ3 and decreased expression of α6β4 are independent, i.e., have different mechanisms.

The expression of integrin αVβ3 is significantly higher in tongue SCC cells than in epithelium cells in normal tissues (Ahmedah et al., 2017). In our previous work we showed the integrin crosstalk event in tongue squamous carcinoma cells Cal27-derived drug resistant cell clone 2B1, obtained by *de novo* expression of integrin αVβ3, i.e., increased amount of integrin αVβ5 in 2B1 cells whose expression was unrelated to integrin αVβ3-mediated anticancer drug resistance to cDDP, MMC and DOX (Stojanović et al., 2016). Therefore, our cell model imitates what might happen during progression of HNSCC. Here, we assessed the adhesome of both cell lines in order to obtain further information on the components of the adhesion complexes and their possible role in regulation of the sensitivity to the anticancer drugs. In conclusion, our major finding is that both Cal27 and 2B1 cells preferentially, but not exclusively, use integrin α6β4 for adhesion forming type II HDs. Integrin α6β4 also provides a physiological role in sensitivity to three different anticancer drugs.

## Conclusion

Since integrins have been implicated in sensitivity to chemotherapy they are therefore potential therapeutic targets, in addition to their already well-described roles as therapeutics in anti-clotting, multiple sclerosis and ulcerative colitis (Desgrosellier and Cheresh, 2010; Seguin et al., 2015; Dickreuter and Cordes, 2017; Raab-Westphal et al., 2017). HDs are important in epithelial cell adhesion, and mutations in any of the six genes encoding the structural components of HDs cause the hereditary skin blistering disorder epidermolysis bullosa (McGrath, 2015). In addition to their adhesion function, HDs may also play an important role in signal transduction (Wang et al., 2020). Finally, HDs play a role in HNSCC progression since its metastatic growth is correlated with upregulation and redistribution of HD components (Herold-Mende et al., 2001). Our results indicate that type II HDs in both Cal27 and 2B1 cells regulate sensitivity to cDDP, MMC and DOX independently of αVβ3. These data also reveal another integrin crosstalk event in Cal27 tongue squamous cell carcinoma cells, i.e., integrin αVβ3-induced upregulation of α6β4, in addition to the previously described integrin crosstalk event, i.e., upregulation of integrin αVβ5 upon *de novo* expression of αVβ3 (Stojanović et al., 2016).

Only those integrins assembled as heterodimers, a process which occurs in the endoplasmic reticulum, are displayed on the cell surface and able to bind their ligands and trigger signaling. Therefore, it is not possible to determine a cell specific integrin repertoire, that is used for cell adhesion and signaling, using transcription analysis (Samaržija et al., 2020). With the development of protocols to permit the isolation and proteomic analysis of IACs it is possible to study the integrins used by particular cell type as well as IAC composition (adhesome) in detail (Humphries et al., 2019). Our data comparing adhesomes of HNSCC cell line Cal27, and its clone 2B1 obtained by *de novo* expression of integrin αVβ3, represent a valuable resource to improve our understanding of the involvement of integrins in sensitivity to chemotherapy as well as integrin crosstalk mechanisms.

## Data Availability

The original contributions presented in the study are publicly available. This data can be found in the ProteomeXchange repository with the dataset identifier: PXD028810.

## Glossary

ACTN1: α-actinin 1
ACTN4: α-actinin 4
AHNAK: neuroblast differentiation-associated protein
ANOVA: related-measure two-way analysis of variance
ANXA1/2: annexins A1 and 2
BM: basement membrane
BP180: BPAG2 or collagen XVII
BP230: dystonin or BPAG1-e
Cal27: tongue squamous carcinoma cells
CAV1: caveolin-1
CD151: tetraspanin
cDDP: cisplatin
CLIC1: chloride intracellular channel 1
COL7A1: collagen VII
CYR61: cysteine-rich 61
DAVID: database for annotation, visualization, and integrated discovery
DDX: DEAD box proteins
DMEM: Dulbecco’s modified Eagle’s medium
DMSO: dimethyl sulfoxide
DOX: doxorubicine
DSG2: desmoglein 2
DSG3: desmoglein 3
DSP: desmoplakin
DTBP: dimethyl 3,3'-dithiobispropionimidate, Wang and Richard's reagent
ECM: extracellular matrix
EDIL-3: EGF-like repeat and discoidin I-like domain-containing protein 3
EDTA: ethylenediaminetetraacetic acid
EMT: epithelial–mesenchymal transition
FA: focal adhesion
FAK: focal adhesion kinase
FBS: foetal bovine serum
FLNA: filamin A
FLNB: filamin B
FN: fibronectin
HEPES: hydroxyethyl piperazineethanesulfonic acid
HD: hemidesmosome
HNSCC: head and neck squamous cell carcinoma
HSPG 2: heparan sulphate proteoglycan 2 or perlecan
HTRA-1: high-temperature requirement serine protease
IAC: integrin adhesion complex
IF: immunofluorescence
ILK: integrin linked kinase
IRM: interference reflection microscopy
ITGA6: integrin subunit α6
ITGB4: integrin subunit β4
ITGB5: integrin subunit β5
KRT-5: keratin 5
KRT-14: keratin 14
LAMA3: laminin subunit α3
LAMB3: laminin subunit β3
LAMC2: laminin subunit γ2
MMC: mitomycin C
MS: mass spectrometry
MTT: 3-(4,5-dimethylthiazol-2-yl)-2,5-diphenyltetrazolium bromide)
NUMA1: nuclear mitotic apparatus protein 1
NUP153: nucleoporin 153
PBS: phosphate-buffered saline
PKP1/2/3: plakophilins 1/2/3
PLAK: junction plakoglobin
PLEC: plectin
PPL: periplakin
PTX: paclitaxel
MT: microtubule
PPI network: protein-protein interaction network
RPL: ribosomal protein large
RPS: ribosomal protein small
SDS-PAGE: sodium dodecyl sulphate-polyacrylamide-gel electrophoresis
Src: nonreceptor tyrosin kinase Src
TEM: transmission electron microscopy
TGFBI: transforming growth factor beta induced
TGF-β: transforming growth factor β
THBS1: thrombospondin-1
TLN1: talin 1
TLN2: talin 2
TNC: tenascin C
VCL: vinculin
VCR: vincristine
VTN: vitronectin
WB: Western blot

## References

[B1] Ahmedah H. Patterson L. Shnyder S. Sheldrake H. (2017). RGD-Binding Integrins in Head and Neck Cancers. Cancers 9 (6), 56. 10.3390/cancers9060056 PMC5483875 28587135

[B2] Albiges-Rizo C. Destaing O. Fourcade B. Planus E. Block M. R. (2009). Actin Machinery and Mechanosensitivity in Invadopodia, Podosomes and Focal Adhesions. J. Cel Sci. 122, 3037–3049. 10.1242/jcs.052704 PMC2767377 19692590

[B3] Ambriović-Ristov A. Gabrilovac J. Čimbora-Zovko T. Osmak M. (2004). Increased Adenoviral Transduction Efficacy in Human Laryngeal Carcinoma Cells Resistant to Cisplatin Is Associated with Increased Expression of Integrin ?v?3 and coxsackie Adenovirus Receptor. Int. J. Cancer 110 (5), 660–667. 10.1002/ijc.20176 15146554

[B4] Apweiler R. Bairoch A. Wu C. H. Barker W. C. Boeckmann B. Ferro S. (2004). UniProt: The Universal Protein Knowledgebase. Nucleic Acids Res. 32 (DATABASE ISS.), D115–D119. Oxford Academic. 10.1093/nar/gky09210.1093/nar/gkh131 14681372 PMC308865

[B5] Atanasova V. S. Russell R. J. Webster T. G. Cao Q. Agarwal P. Lim Y. Z. (2019). Thrombospondin-1 Is a Major Activator of TGF-β Signaling in Recessive Dystrophic Epidermolysis Bullosa Fibroblasts. J. Invest. Dermatol. 139 (7), 1497–1505. Elsevier B.V.e5. 10.1016/j.jid.2019.01.011 30684555

[B6] Aumailley M. Brucknertuderman L. Carter W. Deutzmann R. Edgar D. Ekblom P. (2005). A Simplified Laminin Nomenclature. Matrix Biol. 24 (5), 326–332. 10.1016/j.matbio.2005.05.006 15979864

[B7] Blangy A. (2017). Tensins Are Versatile Regulators of Rho GTPase Signalling and Cell Adhesion. Biol. Cel 109 (3), 115–126. 10.1111/boc.201600053 27748980

[B8] Borradori L. Sonnenberg A. (1999). Structure and Function of Hemidesmosomes: More Than Simple Adhesion Complexes. J. Invest. Dermatol. 112 (4), 411–418. Blackwell Publishing Inc. 10.1046/j.1523-1747.1999.00546.x 10201522

[B9] Boudhraa Z. Bouchon B. Viallard C. D'Incan M. Degoul F. (2016). Annexin A1 Localization and its Relevance to Cancer. Clin. Sci. 130 (4), 205–220. Portland Press Ltd. 10.1042/CS20150415 26769657

[B10] Brozović A. Majhen D. Roje V. Mikac N. Jakopec S. Fritz G. (2008). αvβ3 Integrin-Mediated Drug Resistance in Human Laryngeal Carcinoma Cells Is Caused by Glutathione-Dependent Elimination of Drug-Induced Reactive Oxidative Species. Mol. Pharmacol. 74 (1), 298–306. 10.1124/mol.107.043836 18441044

[B11] Byron A. Askari J. A. Humphries J. D. Jacquemet G. Koper E. J. Warwood S. (2015). A Proteomic Approach Reveals Integrin Activation State-Dependent Control of Microtubule Cortical Targeting. Nat. Commun. 6, 1–14. Nature Publishing Group. 10.1038/ncomms7135 PMC4317495 25609142

[B12] Cen W-N. Pang J.-S. Huang J.-C. Hou J.-Y. Bao W.-G. He R.-Q. (2018). The Expression and Biological Information Analysis of miR-375-3p in Head and Neck Squamous Cell Carcinoma Based on 1825 Samples from GEO, TCGA, and Peer-Reviewed Publications. Pathol. - Res. Pract. 214 (11), 1835–1847. 10.1016/j.prp.2018.09.010 30243807

[B13] Chastney M. R. Lawless C. Humphries J. D. Warwood S. Jones M. C. Knight D. (2020). Topological Features of Integrin Adhesion Complexes Revealed by Multiplexed Proximity Biotinylation. J. Cel. Biol. NLM (Medline) 219 (8), e202003038. 10.1083/jcb.202003038 PMC7401799 32585685

[B14] Choi H. Fermin D. Nesvizhskii A. I. (2008). Significance Analysis of Spectral Count Data in Label-Free Shotgun Proteomics. Mol. Cell Proteomics 7 (12), 2373–2385. 10.1074/mcp.M800203-MCP200 18644780 PMC2596341

[B15] Choi H. Kim S. Fermin D. Tsou C.-C. Nesvizhskii A. I. (2015). QPROT: Statistical Method for Testing Differential Expression Using Protein-Level Intensity Data in Label-free Quantitative Proteomics. J. Proteomics 129, 121–126. Elsevier. 10.1016/J.JPROT.2015.07.036 26254008 PMC4630079

[B16] Coelho B. A. Peterle G. T. Santos M. Agostini L. P. Maia L. L. Stur E. (2015). Keratins 17 and 19 Expression as Prognostic Markers in Oral Squamous Cell Carcinoma. Genet. Mol. Res. 14 (4), 15123–15132. 10.4238/2015.November.24.21 26634475

[B17] Cooper J. Giancotti F. G. (2019). Integrin Signaling in Cancer: Mechanotransduction, Stemness, Epithelial Plasticity, and Therapeutic Resistance. Cancer Cell 35 (3), 347–367. 10.1016/j.ccell.2019.01.007 30889378 PMC6684107

[B18] Costanza B. Rademaker G. Tiamiou A. De Tullio P. Leenders J. Blomme A. (2019). Transforming Growth Factor Beta-Induced, an Extracellular Matrix Interacting Protein, Enhances Glycolysis and Promotes Pancreatic Cancer Cell Migration. Int. J. Cancer 145 (6), 1570–1584. Wiley-Liss Inc. 10.1002/ijc.32247 30834519

[B19] Damiano J. S. Cress A. E. Hazlehurst L. A. Shtil A. A. Dalton W. S. (1999). Cell Adhesion Mediated Drug Resistance (CAM-DR): Role of Integrins and Resistance to Apoptosis in Human Myeloma Cell Lines. Blood 93 (5), 1658–1667. 10.1182/blood.v93.5.1658 10029595 PMC5550098

[B20] Desgrosellier J. S. Cheresh D. A. (2010). Integrins in Cancer: Biological Implications and Therapeutic Opportunities. Nat. Rev. Cancer 10 (1), 9–22. 10.1038/nrc2748 20029421 PMC4383089

[B21] Dickreuter E. Cordes N. (2017). The Cancer Cell Adhesion Resistome: Mechanisms, Targeting and Translational Approaches. Biol. Chem. 398 (7), 721–735. 10.1515/hsz-2016-0326 28002024

[B22] Doncheva N. T. Morris J. H. Gorodkin J. Jensen L. J. (2019). Cytoscape StringApp: Network Analysis and Visualization of Proteomics Data. J. Proteome Res. 18 (2), 623–632. American Chemical Society. 10.1021/acs.jproteome.8b00702 30450911 PMC6800166

[B23] Echarri A. Del Pozo M. A. (2006). Caveolae Internalization Regulates Integrin-Dependent Signaling Pathways. Cell Cycle 5 (19), 2179–2182. 10.4161/cc.5.19.3264 16969102

[B24] Felding-Habermann B. Cheresh D. (1993). Vitronectin and its Receptors. Curr. Opin. Cel Biol. 5 (5), 864–868. 10.1016/0955-0674(93)90036-P 7694604

[B25] Fontao L. Dirrig S. Owaribe K. Kedinger M. Launay J. F. (1997). Polarized Expression of HD1: Relationship with the Cytoskeleton in Cultured Human Colonic Carcinoma Cells. Exp. Cel Res. 231 (2), 319–327. 10.1006/excr.1996.3465 9087173

[B26] Fontao L. Stutzmann J. Gendry P. Launay J. F. (1999). Regulation of the Type II Hemidesmosomal Plaque Assembly in Intestinal Epithelial Cells. Exp. Cel Res. 250 (2), 298–312. 10.1006/excr.1999.4549 10413585

[B27] Gasca J. Flores M. L. Jiménez-Guerrero R. Sáez M. E. Barragán I. Ruíz-Borrego M. (2020). EDIL3 Promotes Epithelial-Mesenchymal Transition and Paclitaxel Resistance through its Interaction with Integrin αVβ3 in Cancer Cells. Cell Death Discov. 6 (1), 86. 10.1038/s41420-020-00322-x 33014430 PMC7494865

[B28] Ginos M. A. Page G. P. Michalowicz B. S. Patel K. J. Volker S. E. Pambuccian S. E. (2004). Identification of a Gene Expression Signature Associated with Recurrent Disease in Squamous Cell Carcinoma of the Head and Neck. Cancer Res. 64 (1), 55–63. 10.1158/0008-5472.CAN-03-2144 14729608

[B29] Goletz S. Zillikens D. Schmidt E. (2017). Structural Proteins of the Dermal-Epidermal Junction Targeted by Autoantibodies in Pemphigoid Diseases. Exp. Dermatol. 26 (12), 1154–1162. 10.1111/exd.13446 28887824

[B30] Han X. Yan S. Weijie Z. Feng W. Liuxing W. Mengquan L. (2014). Critical Role of miR-10b in Transforming Growth Factor-Β1-Induced Epithelial-Mesenchymal Transition in Breast Cancer. Cancer Gene Ther. 21 (2), 60–67. 10.1038/cgt.2013.82 24457988

[B31] Hasegawa M. Cheng J. Maruyama S. Yamazaki M. Abé T. Babkair H. (2016). Differential Immunohistochemical Expression Profiles of Perlecan-Binding Growth Factors in Epithelial Dysplasia, Carcinoma *In Situ*, and Squamous Cell Carcinoma of the Oral Mucosa. Pathol. - Res. Pract. 212 (5), 426–436. 10.1016/j.prp.2016.02.016 26965914

[B32] Hatzfeld M. Magin T. M. (2019). Cross-Talk between Hemidesmosomes and Focal Adhesions: A Primer for Wound Healing, Blistering Skin Disease, and Skin Aging. J. Invest. Dermatol. 139 (9), 1854–1856. 10.1016/j.jid.2019.04.010 31445572

[B33] Hayashi K. Madri J. A. Yurchenco P. D. (1992). Endothelial Cells Interact with the Core Protein of Basement Membrane Perlecan through Beta 1 and Beta 3 Integrins: An Adhesion Modulated by Glycosaminoglycan. J. Cel Biol. 119 (4), 945–959. 10.1083/jcb.119.4.945 PMC2289694 1385448

[B34] Herold-Mende C. Kartenbeck J. Tomakidi P. Bosch F. X. (2001). Metastatic Growth of Squamous Cell Carcinomas Is Correlated with Upregulation and Redistribution of Hemidesmosomal Components. Cell Tissue Res 306 (3), 399–408. 10.1007/s004410100462 11735040

[B35] Hidai C. Zupancic T. Penta K. Mikhail A. Kawana M. Quertermous E. E. (1998). Cloning and Characterization of Developmental Endothelial Locus-1: An Embryonic Endothelial Cell Protein that Binds the αvβ3 Integrin Receptor. Genes Dev. 12 (1), 21–33. 10.1101/gad.12.1.21 9420328 PMC529342

[B36] Hodge K. Have S. T. Hutton L. Lamond A. I. (2013). Cleaning up the Masses: Exclusion Lists to Reduce Contamination with HPLC-MS/MS. J. Proteomics 88, 92–103. 10.1016/j.jprot.2013.02.023 23501838 PMC3714598

[B37] Horton E. R. Byron A. Askari J. A. Ng D. H. J. Millon-Frémillon A. Robertson J. (2015). Definition of a Consensus Integrin Adhesome and its Dynamics during Adhesion Complex Assembly and Disassembly. Nat. Cel Biol 17 (12), 1577–1587. 10.1038/ncb3257 PMC4663675 26479319

[B38] Huang D. W. Sherman B. T. Lempicki R. A. (2009a). Bioinformatics Enrichment Tools: Paths toward the Comprehensive Functional Analysis of Large Gene Lists. Nucleic Acids Res. 37 (1), 1–13. 10.1093/nar/gkn923 19033363 PMC2615629

[B39] Huang D. W. Sherman B. T. Lempicki R. A. (2009b). Systematic and Integrative Analysis of Large Gene Lists Using DAVID Bioinformatics Resources. Nat. Protoc. 4 (1), 44–57. 10.1038/nprot.2008.211 19131956

[B40] Humphries J. D. Byron A. Bass M. D. Craig S. E. Pinney J. W. Knight D. (2009). Proteomic Analysis of Integrin-Associated Complexes Identifies RCC2 as a Dual Regulator of Rac1 and Arf6. Sci. Signal. 2 (87), ra51. 10.1126/scisignal.2000396 19738201 PMC2857963

[B41] Humphries J. D. Chastney M. R. Askari J. A. Humphries M. J. (2019). Signal Transduction via Integrin Adhesion Complexes. Curr. Opin. Cel Biol. 56, 14–21. 10.1016/j.ceb.2018.08.004 30195153

[B42] Hynes R. O. (2002). Integrins: Bidirectional, Allosteric Signaling Machines. Cell 110 (6), 673–687. 10.1016/s0092-8674(02)00971-6 12297042

[B43] Iguchi Y. Ishihara S. Uchida Y. Tajima K. Mizutani T. Kawabata K. (2015). Filamin B Enhances the Invasiveness of Cancer Cells into 3D Collagen Matrices. Cell Struct. Funct. 40 (2), 61–67. 10.1247/csf.15001 25925610

[B44] Jeong D. Ban S. Oh S. Jin Lee S. Yong Park S. Koh Y. W. (2017). Prognostic Significance of EDIL3 Expression and Correlation with Mesenchymal Phenotype and Microvessel Density in Lung Adenocarcinoma. Sci. Rep. 7 (1), 8649. 10.1038/s41598-017-08851-9 28819306 PMC5561239

[B45] Jiang J. Huang L. Yu W. Wu X. Zhou P. Li X. (2012). Overexpression of HTRA1 Leads to Down-Regulation of Fibronectin and Functional Changes in RF/6A Cells and HUVECs. PLoS ONE 7 (10), e46115. 10.1371/journal.pone.0046115 23056244 PMC3466263

[B46] Jones M. C. Humphries J. D. Byron A. Millon-Frémillon A. Robertson J. Paul N. R. (2015). Isolation of Integrin-Based Adhesion Complexes. Curr. Protoc. Cel Biol. 66, 1–21. 10.1002/0471143030.cb0908s66 PMC4402726 25727331

[B47] Kuo J.-C. Han X. Hsiao C.-T. Yates III J. R. III Waterman C. M. (2011). Analysis of the Myosin-II-Responsive Focal Adhesion Proteome Reveals a Role for β-Pix in Negative Regulation of Focal Adhesion Maturation. Nat. Cel Biol 13 (4), 383–393. 10.1038/ncb2216 PMC3279191 21423176

[B48] Kuo J.-C. Han X. Yates J. R. III Waterman C. M. (2012). Isolation of Focal Adhesion Proteins for Biochemical and Proteomic Analysis. Methods Mol. Biol. 757, 297–323. 10.1007/978-1-61779-166-6_19 21909920 PMC4158431

[B49] Lau L. F. (2016). Cell Surface Receptors for CCN Proteins. J. Cel Commun. Signal. 10 (2), 121–127. 10.1007/s12079-016-0324-z PMC4882306 27098435

[B50] Lee J.-H. Jang E. J. Seo H. L. Ku S. K. Lee J. R. Shin S. S. (2014). Sauchinone Attenuates Liver Fibrosis and Hepatic Stellate Cell Activation through TGF-β/Smad Signaling Pathway. Chemico-Biological Interactions 224, 58–67. 10.1016/j.cbi.2014.10.005 25451574

[B51] Litjens S. H. M. de Pereda J. M. Sonnenberg A. (2006). Current Insights into the Formation and Breakdown of Hemidesmosomes. Trends Cel Biol. 16 (7), 376–383. 10.1016/j.tcb.2006.05.004 16757171

[B52] Lock J. G. Baschieri F. Jones M. C. Humphries J. D. Montagnac G. Strömblad S. (2019). Clathrin-Containing Adhesion Complexes. J. Cel Biol. 218 (7), 2086–2095. 10.1083/jcb.201811160 PMC6605790 31208994

[B53] Lock J. G. Jones M. C. Askari J. A. Gong X. Oddone A. Olofsson H. (2018). Reticular Adhesions Are a Distinct Class of Cell-Matrix Adhesions that Mediate Attachment during Mitosis. Nat. Cel Biol 20 (11), 1290–1302. 10.1038/s41556-018-0220-2 30361699

[B54] Loschke F. Homberg M. Magin T. M. (2016). Keratin Isotypes Control Desmosome Stability and Dynamics through PKCα. J. Invest. Dermatol. 136 (1), 202–213. 10.1038/JID.2015.403 26763440

[B55] Majhen D. Stojanović N. Vukić D. Pichon C. Leduc C. Osmak M. (2014). Increased Adenovirus Type 5 Mediated Transgene Expression Due to RhoB Down-Regulation. PLoS ONE 9 (1), e86698. 10.1371/journal.pone.0086698 24466204 PMC3899303

[B56] McGrath J. A. (2015). Recently Identified Forms of Epidermolysis Bullosa. Ann. Dermatol. 27 (6), 658–666. 10.5021/ad.2015.27.6.658 26719633 PMC4695416

[B57] Meireles Da Costa N. Mendes F. A. Pontes B. Nasciutti L. E. Ribeiro Pinto L. F. Palumbo Júnior A. (2021). Potential Therapeutic Significance of Laminin in Head and Neck Squamous Carcinomas. Cancers 13, 1890. 10.3390/cancers13081890 33920762 PMC8071176

[B58] Midwood K. S. Chiquet M. Tucker R. P. Orend G. (2016). Tenascin-C at a Glance. J. Cel Sci. 129 (23), 4321–4327. Company of Biologists Ltd. 10.1242/jcs.190546 27875272

[B59] Myllymäki S.-M. Kämäräinen U.-R. Liu X. Cruz S. P. Miettinen S. Vuorela M. (2019). Assembly of the β4-Integrin Interactome Based on Proximal Biotinylation in the Presence and Absence of Heterodimerization*. Mol. Cell Proteomics 18 (2), 277–293. 10.1074/mcp.RA118.001095 30404858 PMC6356083

[B60] Nesvizhskii A. I. Keller A. Kolker E. Aebersold R. (2003). A Statistical Model for Identifying Proteins by Tandem Mass Spectrometry. Anal. Chem. 75 (17), 4646–4658. 10.1021/ac0341261 14632076

[B61] Nishimori T. Tomonaga T. Matsushita K. Oh-Ishi M. Kodera Y. Maeda T. (2006). Proteomic Analysis of Primary Esophageal Squamous Cell Carcinoma Reveals Downregulation of a Cell Adhesion Protein, Periplakin. Proteomics 6 (3), 1011–1018. 10.1002/pmic.200500262 16400690

[B62] Owaribe K. Kartenbeck J. Stumpp S. Magin T. M. Krieg T. Diaz L. A. (1990). The Hemidesmosomal Plaque. Differentiation 45 (3), 207–220. 10.1111/j.1432-0436.1990.tb00475.x 2090522

[B63] Paradžik M. Humphries J. D. Stojanović N. Nestić D. Majhen D. Dekanić A. (2020). KANK2 Links αVβ5 Focal Adhesions to Microtubules and Regulates Sensitivity to Microtubule Poisons and Cell Migration. Front. Cel Dev. Biol. 8, 1–17. 10.3389/fcell.2020.00125 PMC7063070 32195252

[B64] Peretti M. Angelini M. Savalli N. Florio T. Yuspa S. H. Mazzanti M. (2015). Chloride Channels in Cancer: Focus on Chloride Intracellular Channel 1 and 4 (CLIC1 and CLIC4) Proteins in Tumor Development and as Novel Therapeutic Targets. Biochim. Biophys. Acta (Bba) - Biomembranes 1848 (10 Pt B), 2523–2531. 10.1016/j.bbamem.2014.12.012 25546839

[B65] Pora A. Yoon S. Windoffer R. Leube R. E. (2019). Hemidesmosomes and Focal Adhesions Treadmill as Separate but Linked Entities during Keratinocyte Migration. J. Invest. Dermatol. 139 (9), 1876–1888. e4. 10.1016/j.jid.2019.03.1139 30951704

[B66] Pouliot N. Kusuma N. (2013). Laminin-511Cell Adhesion and Migration. Cell Adhes. Migration 7 (1), 142–149. 10.4161/cam.22125 PMC3544778 23076212

[B67] Quick Q. (2018). Microtubule-actin Crosslinking Factor 1 and Plakins as Therapeutic Drug Targets. Int. J. Mol. Sci. MDPI AG 19 (2), 368. 10.3390/ijms19020368 PMC5855590 29373494

[B68] Raab-Westphal S. Marshall J. Goodman S. Raab-Westphal S. Marshall J. F. Goodman S. L. (2017). Integrins as Therapeutic Targets: Successes and Cancers. Cancers 9 (12), 110. 10.3390/cancers9090110 PMC5615325 28832494

[B69] Ramovs V. te Molder L. Sonnenberg A. (2017). The Opposing Roles of Laminin-Binding Integrins in Cancer. Matrix Biol. 57-58 (58), 213–243. 10.1016/j.matbio.2016.08.007 27562932

[B70] Samaržija I. Dekanić A. Humphries J. D. Paradžik M. Stojanović N. Humphries M. J. (2020). Integrin Crosstalk Contributes to the Complexity of Signalling and Unpredictable Cancer Cell Fates. Cancers 12 (7), 1910–1926. 10.3390/cancers12071910 PMC7409212 32679769

[B71] Schiller H. B. Friedel C. C. Boulegue C. Fässler R. (2011). Quantitative Proteomics of the Integrin Adhesome Show a Myosin II-Dependent Recruitment of LIM Domain Proteins. EMBO Rep. 12 (3), 259–266. 10.1038/embor.2011.5 21311561 PMC3059911

[B72] Seguin L. Desgrosellier J. S. Weis S. M. Cheresh D. A. (2015). Integrins and Cancer: Regulators of Cancer Stemness, Metastasis, and Drug Resistance. Trends Cel Biol. 25 (4), 234–240. 10.1016/j.tcb.2014.12.006 PMC4380531 25572304

[B73] Shannon P. Markiel A. Ozier O. Baliga N. S. Wang J. T. Ramage D. (2003). Cytoscape: A Software Environment for Integrated Models of Biomolecular Interaction Networks. Genome Res. 13 (11), 2498–2504. 10.1101/gr.1239303 14597658 PMC403769

[B74] Stojanović N. Brozovic A. Majhen D. Bosnar M. H. Fritz G. Osmak M. (2016). Integrin αvβ3 Expression in Tongue Squamous Carcinoma Cells Cal27 Confers Anticancer Drug Resistance through Loss of pSrc(Y418). Biochim. Biophys. Acta (Bba) - Mol. Cel Res. 1863 (8), 1969–1978. 10.1016/j.bbamcr.2016.04.019 27108184

[B75] Supek F. Bošnjak M. Škunca N. Šmuc T. (2011). Revigo Summarizes and Visualizes Long Lists of Gene Ontology Terms. PLoS ONE 6 (7), e21800. 10.1371/journal.pone.0021800 21789182 PMC3138752

[B76] Szklarczyk D. Gable A. L. Lyon D. Junge A. Wyder S. Huerta-Cepas J. (2019). STRING V11: Protein-Protein Association Networks with Increased Coverage, Supporting Functional Discovery in Genome-Wide Experimental Datasets. Nucleic Acids Res. 47 (D1), D607–D613. 10.1093/nar/gky1131 30476243 PMC6323986

[B77] Te Molder L. Juksar J. Harkes R. Wang W. Kreft M. Sonnenberg A. (2019). Tetraspanin CD151 and Integrin α3β1 Contribute to the Stabilization of Integrin α6β4-Containing Cell-Matrix Adhesions. J. Cel. Sci. 132 (19), jcs235366. 10.1242/jcs.235366 31488507

[B78] Todorović V. Desai B. V. Eigenheer R. A. Yin T. Amargo E. V. Mrksich M. (2010). Detection of Differentially Expressed Basal Cell Proteins by Mass Spectrometry. Mol. Cell Proteomics 9 (2), 351–361. 10.1074/mcp.M900358-MCP200 19955077 PMC2830845

[B79] Turashvili G. Bouchal J. Baumforth K. Wei W. Dziechciarkova M. Ehrmann J. (2007). Novel Markers for Differentiation of Lobular and Ductal Invasive Breast Carcinomas by Laser Microdissection and Microarray Analysis. BMC Cancer 7, 55. 10.1186/1471-2407-7-55 17389037 PMC1852112

[B80] Uematsu J. Nishizawa Y. Sonnenberg A. Owaribe K. (1994). Demonstration of Type II Hemidesmosomes in a Mammary Gland Epithelial Cell Line, BMGE-H1. J. Biochem. 115 (3), 469–476. 10.1093/oxfordjournals.jbchem.a124361 8056759

[B81] Walko G. Castañón M. J. Wiche G. (2015). Molecular Architecture and Function of the Hemidesmosome. Cel Tissue Res 360 (3), 529–544. 10.1007/s00441-014-2061-z PMC4452579 26017636

[B82] Wang C. Lin C. F. (2014). Annexin A2: Its Molecular Regulation and Cellular Expression in Cancer Development. Dis. Markers. 10.1155/2014/308976 PMC3925611 24591759

[B83] Wang W. Zuidema A. Te Molder L. Nahidiazar L. Hoekman L. Schmidt T. (2020). Hemidesmosomes Modulate Force Generation via Focal Adhesions. J. Cel. Biol. 219 (2), e201904137. 10.1083/jcb.201904137 PMC7041674 31914171

[B84] Ween M. P. Oehler M. K. Ricciardelli C. (2012). Transforming Growth Factor-Beta-Induced Protein (TGFBI)/(βig-H3): A Matrix Protein with Dual Functions in Ovarian Cancer. Int. J. Mol. Sci. 13 (8), 10461–10477. Multidisciplinary Digital Publishing Institute (MDPI). 10.3390/ijms130810461 22949874 PMC3431872

[B85] Wesley T. Berzins S. Kannourakis G. Ahmed N. (2021). The Attributes of Plakins in Cancer and Disease: Perspectives on Ovarian Cancer Progression, Chemoresistance and Recurrence. Cell Commun Signal 19 (1), 55. 10.1186/s12964-021-00726-x 34001250 PMC8127266

[B86] Willett M. Pollard H. J. Vlasak M. Morley S. J. (2010). Localization of Ribosomes and Translation Initiation Factors to Talin/β3-Integrin-Enriched Adhesion Complexes in Spreading and Migrating Mammalian Cells. Biol. Cel 102 (5), 265–276. 10.1042/BC20090141 19929852

[B87] Winograd-Katz S. E. Fässler R. Geiger B. Legate K. R. (2014). The Integrin Adhesome: From Genes and Proteins to Human Disease. Nat. Rev. Mol. Cel Biol 15 (4), 273–288. 10.1038/nrm3769 24651544

[B88] Woychek A. Kligys K. Hopkinson S. B. Jones J. C. R. (2019). The 3′UTR of the α6 Integrin Message Regulates Localization of α6β4 Integrin Heterodimers. Biochem. Biophysical Res. Commun. 513 (1), 8–14. 10.1016/j.bbrc.2019.03.116 30922568

[B89] Yadav A. Kumar B. Datta J. Teknos T. N. Kumar P. (2011). IL-6 Promotes Head and Neck Tumor Metastasis by Inducing Epithelial-Mesenchymal Transition via the JAK-STAT3-SNAIL Signaling Pathway. Mol. Cancer Res. 9 (12), 1658–1667. 10.1158/1541-7786.MCR-11-0271 21976712 PMC3243808

[B90] Yamashiro Y. Thang B. Q. Ramirez K. Shin S. J. Kohata T. Ohata S. (2020). Matrix Mechanotransduction Mediated by Thrombospondin-1/integrin/YAP in the Vascular Remodeling. Proc. Natl. Acad. Sci. USA 117 (18), 9896–9905. 10.1073/pnas.1919702117 32321834 PMC7211957

[B91] Yuh D.-Y. Maekawa T. Li X. Kajikawa T. Bdeir K. Chavakis T. (2020). The Secreted Protein DEL-1 Activates a β3 Integrin-FAK-Erk1/2-RUNX2 Pathway and Promotes Osteogenic Differentiation and Bone Regeneration. J. Biol. Chem. 295 (21), 7261–7273. American Society for Biochemistry and Molecular Biology Inc. 10.1074/jbc.RA120.013024 32280065 PMC7247308

[B92] Zaidel-Bar R. Itzkovitz S. Ma'ayan A. Iyengar R. Geiger B. (2007). Functional Atlas of the Integrin Adhesome. Nat. Cel Biol 9 (8), 858–867. 10.1038/ncb0807-858 PMC2735470 17671451

[B93] Zamir E. Katz B. Z. Aota S. Yamada K. M. Geiger B. Kam Z. (1999). Molecular Diversity of Cell-Matrix Adhesions. J. Cel Sci 112 (Pt 11), 1655–1669. 10.1242/jcs.112.11.1655 10318759

[B94] Zuidema A. Wang W. Sonnenberg A. (2020). Crosstalk Between Cell Adhesion Complexes in Regulation of Mechanotransduction. BioEssays 42, 2000119. 10.1002/bies.202000119 32830356

