# Supplementary material for: The Tongue Squamous Carcinoma Cell Line Cal27 Primarily Employs Integrin α6β4-Containing Type II Hemidesmosomes for Adhesion Which Contribute to Anticancer Drug Sensitivity
Source: Front Cell Dev Biol. 2021 Dec 16;9:786758. doi: 10.3389/fcell.2021.786758 (PMC8716755; doi:10.3389/fcell.2021.786758)
Supplement: Supplementary file 2 [file DataSheet1.pdf]

Supplementary Material

Supplementary Methods

Flow cytometry (related to Supplementary Figure 2A)

To analyze the expression of integrin subunit α6 following integrin subunit β4 knockdown, flow cytometry was performed. Briefly, 48 h upon transient β4-specific siRNA transfection adherent cells grown in tissue culture dishes were detached by EDTA (Invitrogen, United States) and washed twice with PBS. Membrane fluorescence staining was performed using unlabeled integrin subunit α6-specific monoclonal primary antibody (1 h, 4°C, Mab listed in Supplementary Table S1), followed by incubation with FITC-conjugated anti-mouse antibody (30 min, 4°C). Isotype control samples were incubated with mouse IgG1 followed by FITC-conjugated anti-mouse antibody. Data acquisition was performed on FACSCalibur using BD CellQuest software package (all BD Biosciences, United States). Data were analyzed with FCS Express 3 (De Novo Software, United States) software.

Supplementary Figures

Supplementary Figure S1

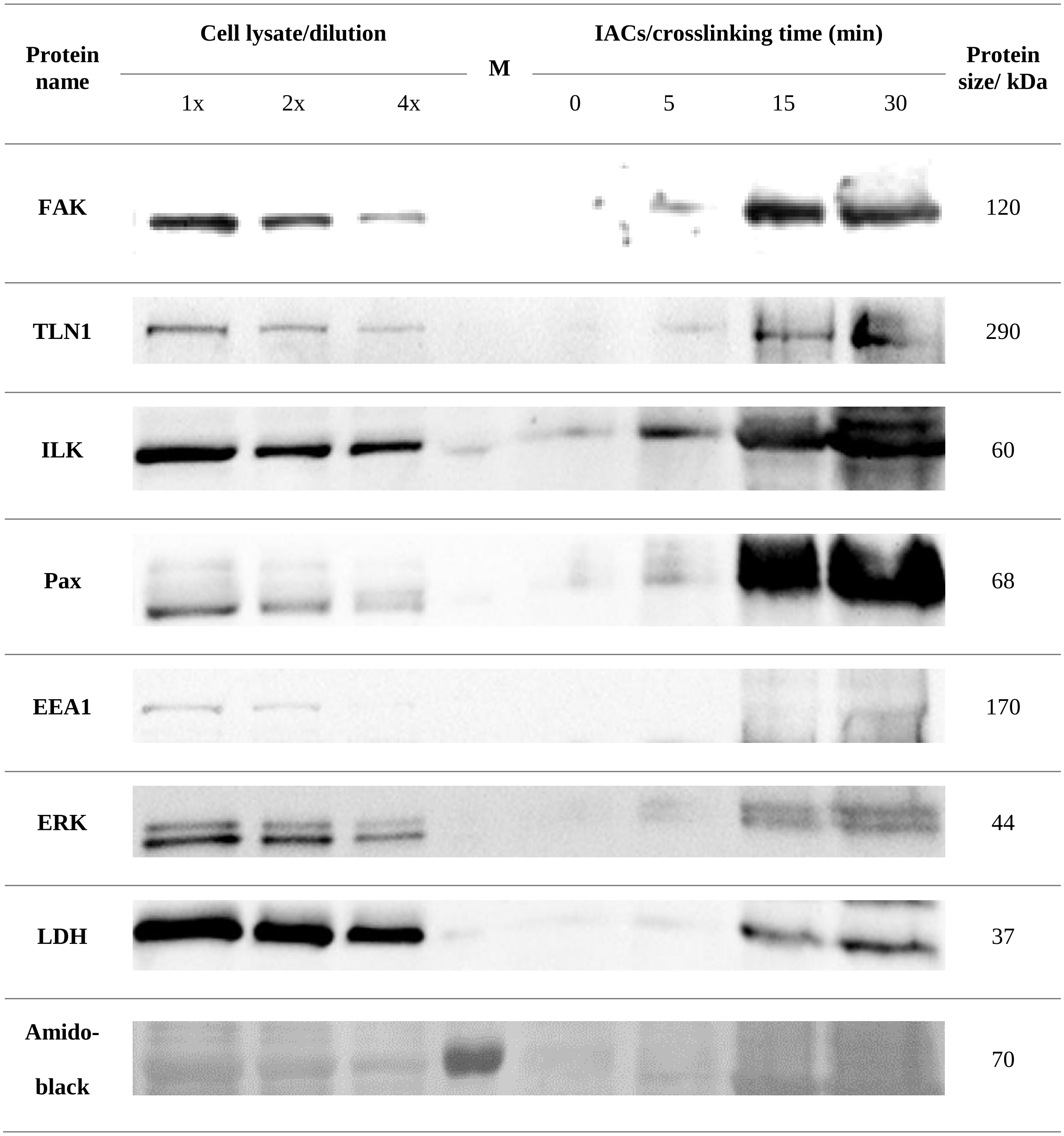

Supplementary Figure S1. Optimization of crosslinking duration in Cal27 cells. The optimal crosslinking duration of 10 minutes was selected based on the WB analysis of marker IAC components, FAK, TLN1, integrin linked kinase (ILK) and paxillin (Pax). WB analysis of components that do not classically compartmentalize with adhesion complexes, early endosome antigen 1 (EEA1), extracellular signal-regulated kinase (ERK) and lactate dehydrogenase (LDH), was used to estimate co-purifying contaminants.

Supplementary Figure S2

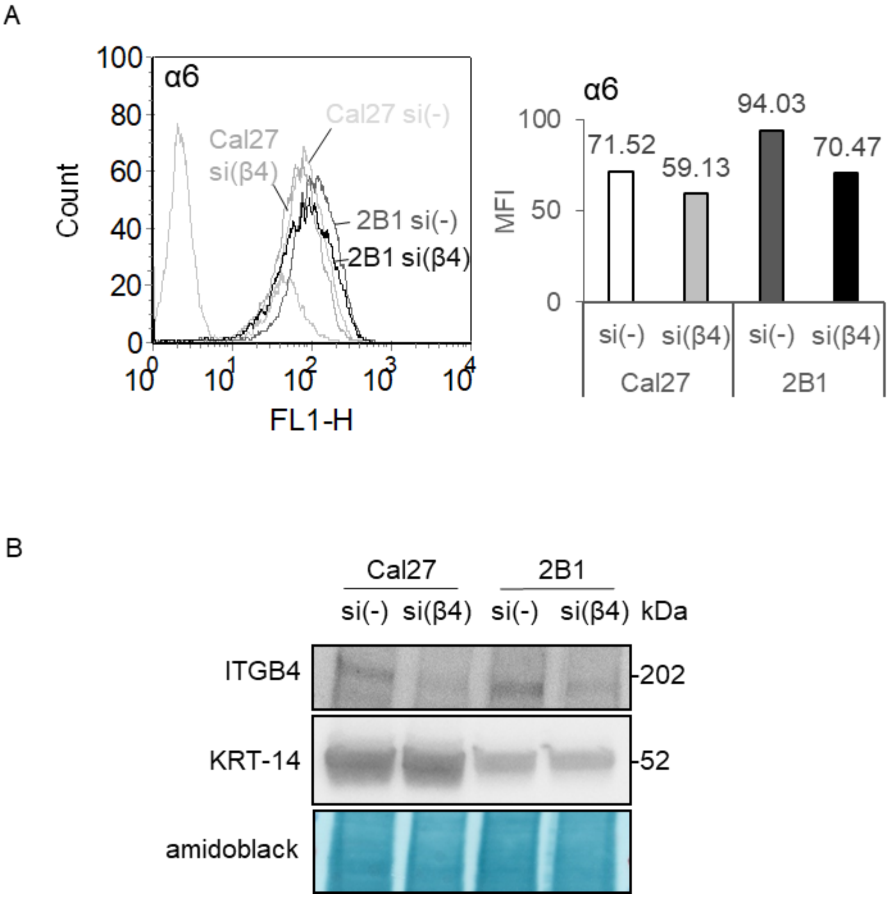

Supplementary Figure S2. Knockdown of integrin β4 in Cal27 and 2B1 cells reduces the expression of integrin subunit α6, but does not influence KRT14 expression in either cell line. (A) Surface expression of integrin subunit α6 in Cal27 or 2B1 cells was analyzed by indirect flow cytometry 48 hours after transfection with integrin subunit β4-specific siRNA (si(β4)) and compared with cells transfected with control siRNA (si(-)). Representative histograms and mean fluorescence intensities (MFI) are shown. (B) Western blot analysis of β4 and KRT14 expression in Cal27 and 2B1 cells transfected with control (si(-)) or β4 specific siRNA (si(β4)). Forty-eight hours after transfection total cell lysates were collected and WB analysis was performed. The results presented are representative of four independent experiments yielding similar results. ITGB4, integrin subunit β4; KRT-14, keratin 14

Supplementary Figure S3

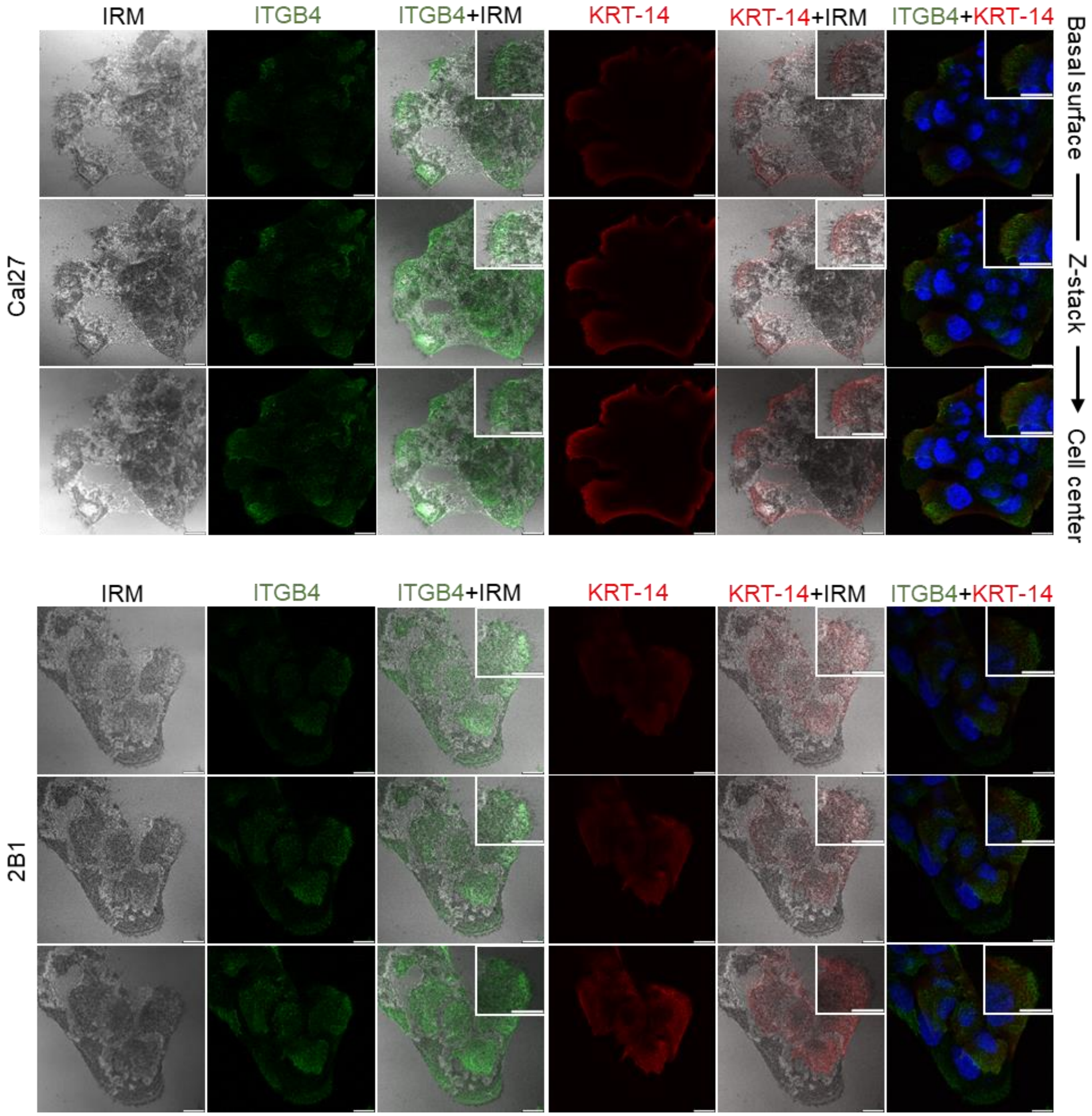

Supplementary Figure S3. KRT-14 is superimposed by integrin subunit β4 in both Cal27 or 2B1 cells but is distributed differently in Cal27 as compared to 2B1. Confocal z stack images of Cal27 and 2B1 cells. Forty-eight hours after seeding on coverslips, cells were fixed, permeabilized, incubated with antibodies against integrin anti-β4 (ITGB4) antibody followed by Alexa-Fluor 488-conjugated antibody (green) and KRT-14 antibody followed by Alexa-Fluor 564-conjugated antibody (red). Nuclei were stained with DAPI (blue). IRM images were taken and analysis was performed using TCS SP8 Leica. Scale bar = 10 μm.

Supplementary Figure S4

Figure 2C

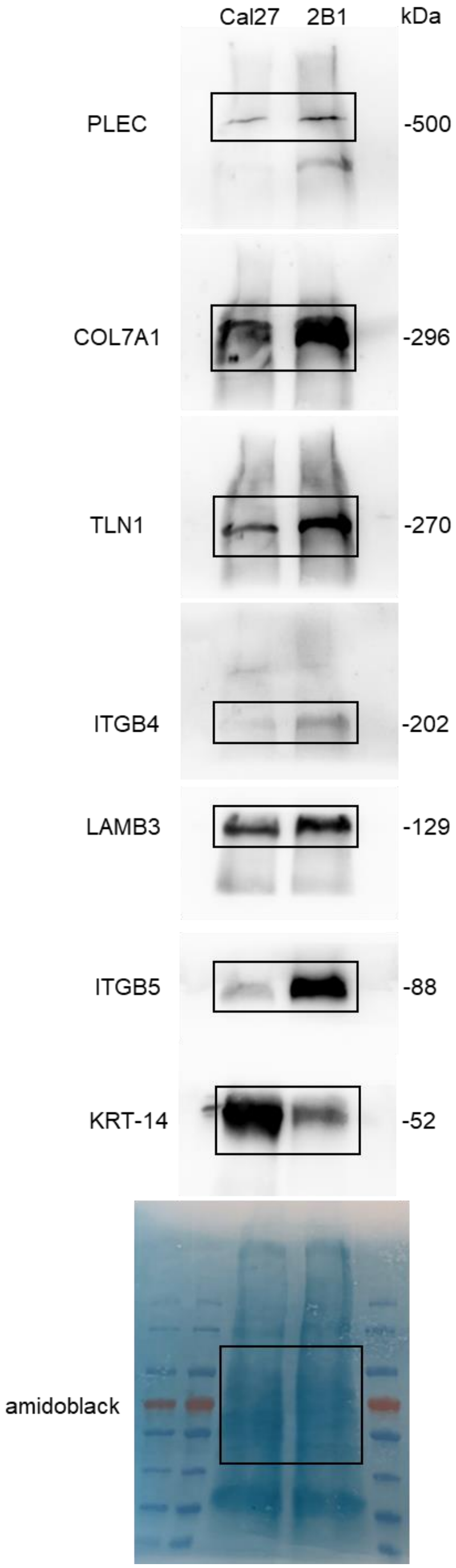

Supplementary Figure S4. Full images of the blots in Figure 2C. Images were obtained using iBright CL1000, which directly scanned membranes developed with ECL reagents.

Supplementary Figure S5

Supplementary Figure S2B

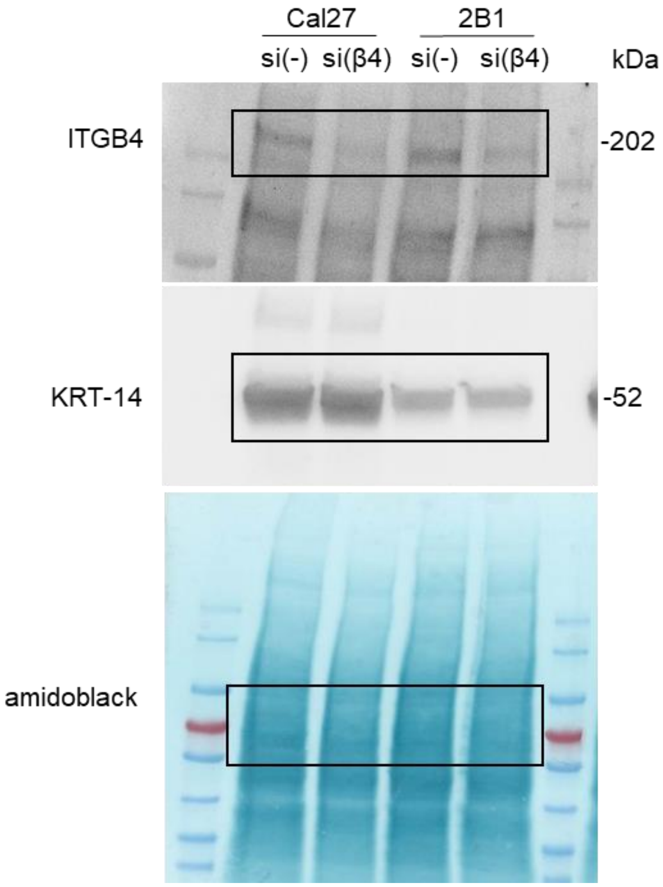

Supplementary Figure S5. Full images of the blots in Supplementary Figure S2B. Images were obtained using iBright CL1000, which directly scanned membranes developed with ECL reagents.

Supplementary Table

Supplementary Table 1 List of primary antibodies used in this study.

			Dilution			
**Antibody**	**Cat No.**	**Manufacturer**	**WB**	**IF**	**FC**	**Species**
IgG1 isotype control from murine myeloma	M5284	Sigma-Aldrich, USA			1:50	mouse
Anti-CD49f Antibody, clone 4F10 (Integrin subunit α6, ITGA6)	CBL458	Merck Millipore, Germany		1:100	1:60	mouse
Anti-Integrin β4 antibody, clone 3E1	MAB1964	Merck Millipore, Germany	1:500	1:200		mouse
Integrin β5 (D24A5)	3629	Cell Signaling, USA	1:1000			rabbit
Talin-1 antibody, clone 97H6	MCA4770GA	Bio-Rad, USA	1:1000			mouse
Plectin (10F6)	sc-33649	Santa Cruz, USA	1:200			mouse
Anti-LAMB3 antibody	AMAB91161	Sigma-Aldrich, USA	1:500			mouse
Anti-COL7A1 antibody	HPA042420	Sigma-Aldrich, USA	1:750			rabbit
Purified anti-Keratin 14 Antibody (clone Poly19053)	905303	BioLegend, USA	1:30 000	1:300		rabbit
Recombinant Anti-FAK antibody [EP695Y]	ab40794	Abcam, USA	1:1000			rabbit
ILK1 Antibody	3862S	Cell Signaling Technology, USA	1:1000			rabbit
Recombinant Anti-Paxillin antibody [Y113]	ab32084	Abcam, USA	1:7500			rabbit
EEA1 Antibody	2411	Cell Signaling Technology, USA	1:1000			rabbit
ERK 1 Antibody (K-23)	sc94	Santa Cruz Biotechnology USA	1:1000			rabbit
LDH Antibody (H-160)	sc33781	Santa Cruz Biotechnology, USA	1:400			rabbit
